# Galactosaminogalactan, a New Immunosuppressive Polysaccharide of *Aspergillus fumigatus*

**DOI:** 10.1371/journal.ppat.1002372

**Published:** 2011-11-10

**Authors:** Thierry Fontaine, Aurélie Delangle, Catherine Simenel, Bernadette Coddeville, Sandra J. van Vliet, Yvette van Kooyk, Silvia Bozza, Silvia Moretti, Flavio Schwarz, Coline Trichot, Markus Aebi, Muriel Delepierre, Carole Elbim, Luigina Romani, Jean-Paul Latgé

**Affiliations:** 1 Unité des Aspergillus, Institut Pasteur, Paris, France; 2 Unité de Résonance Magnétique Nucléaire des Biomolécules, CNRS URA 2185, Institut Pasteur, Paris, France; 3 Laboratoire de Glycobiologie Structurale et Fonctionnelle, UMR 8576 CNRS, Université des sciences et Technologies de Lille Flandres-Artois, Villeneuve d'Ascq, France; 4 Department of Molecular Cell Biology and Immunology, VU University Medical Center, Amsterdam, The Netherlands; 5 Department of Experimental Medicine and Biochemical Sciences, University of Perugia, Perugia, Italy; 6 Institute of Microbiology, ETH Hönggerberg, Zürich, Switzerland; 7 Université Pierre et Marie Curie – Paris 6, UMR-S 945 Immunité et Infection, Faculté de Médecine Pitié Salpétrière, Paris, France; Albert Einstein College of Medicine, United States of America

## Abstract

A new polysaccharide secreted by the human opportunistic fungal pathogen *Aspergillus fumigatus* has been characterized. Carbohydrate analysis using specific chemical degradations, mass spectrometry, ^1^H and ^13^C nuclear magnetic resonance showed that this polysaccharide is a linear heterogeneous galactosaminogalactan composed of α1-4 linked galactose and α1-4 linked N-acetylgalactosamine residues where both monosacharides are randomly distributed and where the percentage of galactose per chain varied from 15 to 60%. This polysaccharide is antigenic and is recognized by a majority of the human population irrespectively of the occurrence of an *Aspergillus* infection. GalNAc oligosaccharides are an essential epitope of the galactosaminogalactan that explains the universal antibody reaction due to cross reactivity with other antigenic molecules containing GalNAc stretches such as the N-glycans of *Campylobacter jejuni*. The galactosaminogalactan has no protective effect during *Aspergillus* infections. Most importantly, the polysaccharide promotes fungal development in immunocompetent mice due to its immunosuppressive activity associated with disminished neutrophil infiltrates.

## Author Summary

*Aspergillus fumigatus* is an opportunistic human fungal pathogen that causes a wide range of diseases including allergic reactions and local or systemic infections such as invasive pulmonary aspergillosis that has emerged in the recent years as a leading cause of infection related mortality among immunocompromised patients. Polysaccharides from the fungal cell wall play essential biological functions in the fungal cell biology and in host-pathogen interactions. Indeed, it has been shown that polysaccharides can modulate the human immune response; some of them (β-glucan and α-glucans) having a protective effect against *Aspergillus* infection. We report here the purification and chemical characterization of a new antigenic polysaccharide (galactosaminogalactan) produced by *A. fumigatus*. This polymer is secreted during infection. In murine models of aspergillosis, this galactosaminogalactan is not protective but it is immunosuppressive and favors *A. fumigatus* infection. Particularly it induces the apoptotic death of neutrophils that are the phagocytes playing an essential role in the killing of fungal pathogens.

## Introduction

*Aspergillus fumigatus* is an opportunistic human fungal pathogen that causes a wide range of diseases including allergic reactions and local or systemic infections such as invasive pulmonary aspergillosis (IA) that has emerged in recent years as a leading cause of infection-related mortality among immunocompromised patients [1], [2]. The innate immune system provides the first line of defense against *A. fumigatus* with macrophages and neutrophils that sense, phagocytose and kill conidia and hyphae through the production of anti-microbial agents. Later, antigen presenting cells initiate an adaptative response activating various populations of T-helper cells that impact differently on the evolution of the disease [3], [4]. Because of its external localisation, and specific composition, the cell wall represents a specific target for recognition and specific interaction with the host immune cells. The cell wall of *A. fumigatus* is mainly composed of branched β1-3glucans, α1-3glucans, chitin, β1-3/1-4 glucan and galactomannan [5]. These constitutive polysaccharides have been shown to induce specific immune responses from the host. For example in murine models of aspergillosis, α1-3glucan and β1-3glucan chains induce a protective response through the activation of Th1 and Th17 or Treg responses [4] whereas galactomannan favours the disease through the activation of the Th2/Th17 response. In other medically important fungi, capsular and cell wall polysaccharides and especially mannan and β-glucans also induce an immune response that either favours or inhibits fungal infection [6], [7], [8], [9].

During growth *in vitro* in aerial conditions or *in vivo* in the lung tissues, the mycelium of *A. fumigatus* is covered by a polysaccharide-rich extracellular matrix (ECM) that because of its outer position, plays a major role in the interaction with the host immune cells [10], [11]. The ECM contains α1-3glucan and galactomannan that are two of the major cell wall polysaccharides, recognised by T cells. A third galactosamine-rich polysaccharide has been now identified in the ECM. Although the presence of such cell wall associated polysaccharide was noticed 20 years ago [12], [13], its structural analysis has not been investigated to date. The present report shows that this polysaccharide is a linear heterogenous chain constituted by α1-4 linked galactose and α1-4 linked N-acetylgalactosamine residues. Most interestingly, the analysis of the immune response towards this polysaccharide shows that it is immunosuppressive and favors *A. fumigatus* infection.

## Results

### A galactosaminogalactan is secreted by the mycelium of *A. fumigatus*

**Figure 1:**
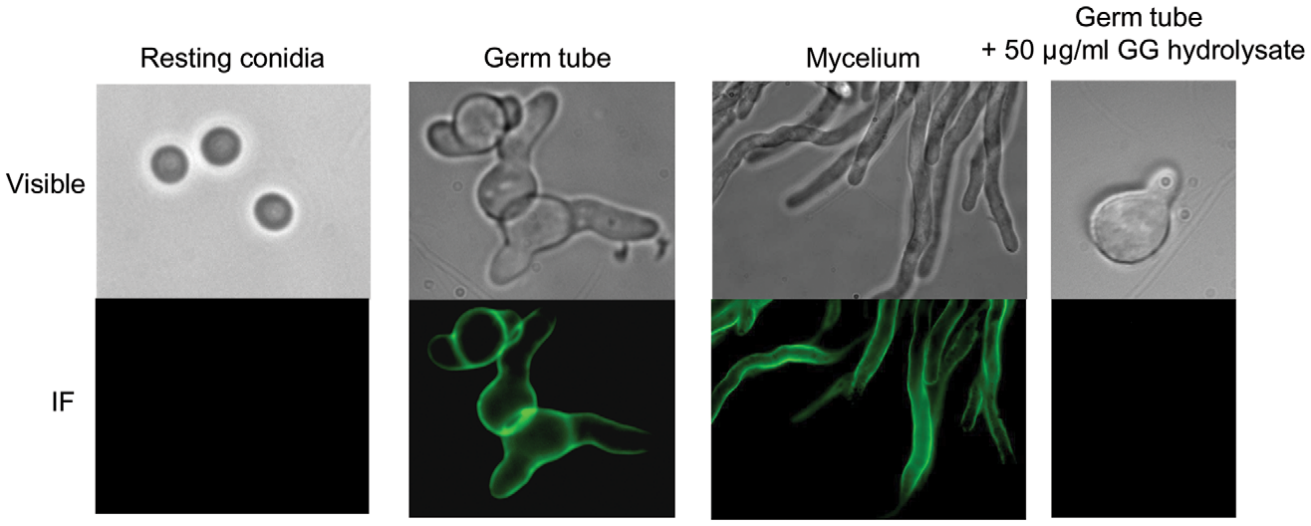
Detection of the galactosaminogalactan by immunofluorescence on resting, germinated conidia and on mycelium. The specificity of the cell wall labelling was confirmed by the full inhibition of the labelling with the anti-GG MAb recognition seen after the MAb was incubated with GalNAc oligosaccharides obtained by HCl hydrolysis galactosaminogalactan (last panel on the right).

The culture filtrate of *A. fumigatus* was precipitated by 70% ethanol. In our experimental conditions, an amount of 80 mg of ethanol precipitate was recovered per g of mycelial dry weight. The incubation of the ethanol precipitate of the culture filtrate of *A. fumigatus* for 1 h in a 150 mM NaCl aqueous solution resulted in the solubilisation of glycoproteins and galactomannan. The NaCl-insoluble material represented 43+/−8% of the ethanol precipitate. The remaining insoluble material was separated in two fractions based on their solubility in 8 M urea. The urea-soluble material (SGG, urea soluble galactosaminogalactan) accounted for 30+/− 4% of the total ethanol precipitate whereas the urea-insoluble material (PGG, urea insoluble galactosaminogalactan) represented 13+/− 6% of the total ethanol precipitate. Gas liquid chromatography (GC) analysis of both fractions showed that they were exclusively composed of galactosamine and galactose with ratios of 60/40 and 15/85 in SGG and PGG respectively. Nitrous deamination of native polysaccharide did not solubilise the polysaccharide and did not produce anhydrotalose showing that all galactosamine residues were N-acetylated (not shown). GC analysis showed that the galactosaminogalactan was absent in resting conidia but was present in the cell wall of mycelium from both solid and liquid cultures and in different media (not shown). Immunofluorescence with specific anti-GG mAb confirmed that GG was not present on the surface of resting conidia. In contrast, a positive detection was seen in the cell wall as soon as the conidium germinates (Fig. 1). This result indicated that part of the galactosaminogalactan was not secreted and remained strongly associated with the cell wall. The amount of cell wall bound galactosaminogalactan was equivalent to the amount recovered in the culture medium (data not shown).

### Structural analysis of GG

GC analysis of permethylated GG revealed only two methyl ethers: 2,3,6-tri-O-methyl-galactitol and 3,6-di-O-methyl-N-acetylgalactosaminitol (Fig. S1), indicating the substitution in position 4 of both monosaccharides. The absence of methylether from non-reducing end sugar or disubstituted monosaccharide indicated that the galactosaminogalactan was an unbranched linear polysaccharide. The apparent Mr estimated by gel filtration chromatography after the carboxymethylation of the GG fraction was in agreement with methylation data. The galactosaminogalactan was eluted as a polydisperse homogenous polymer between 10 and 1000 kDa with a median size of 100 kDa (Fig. S2). The 1D ^1^H and 2D ^1^H, ^13^C nuclear magnetic resonance (NMR) spectra of carboxymethylated GG fraction exhibited two main signals in the sugar anomeric region at 5.003/103.07 and 5.287/99.07 ppm compatible with α-anomers (Fig. S3). NMR data showed downfield shifts for the carbone-4 of both sugar residues, indicating their 4-O substitution and their pyranose configuration, which were in agreement with the methylation data.

In order to elucidate the repartition of each monosaccharide on the main polysaccharidic chain, two specific chemical degradations of both galactosaminogalactan fractions (PGG and SGG) were undertaken: periodate oxidation that degraded 4-O-substituted galactose residues and N-de-acetylation/nitrous deamination that degraded hexosamine residues. Solubilised fractions were separated by gel filtration on HW40S column and chemically analysed by methylation, GC-Mass spectrometry (GC-MS), Matrix-assisted laser desorption-Time of flight (MALDI-TOF) and NMR.

**Figure 2:**
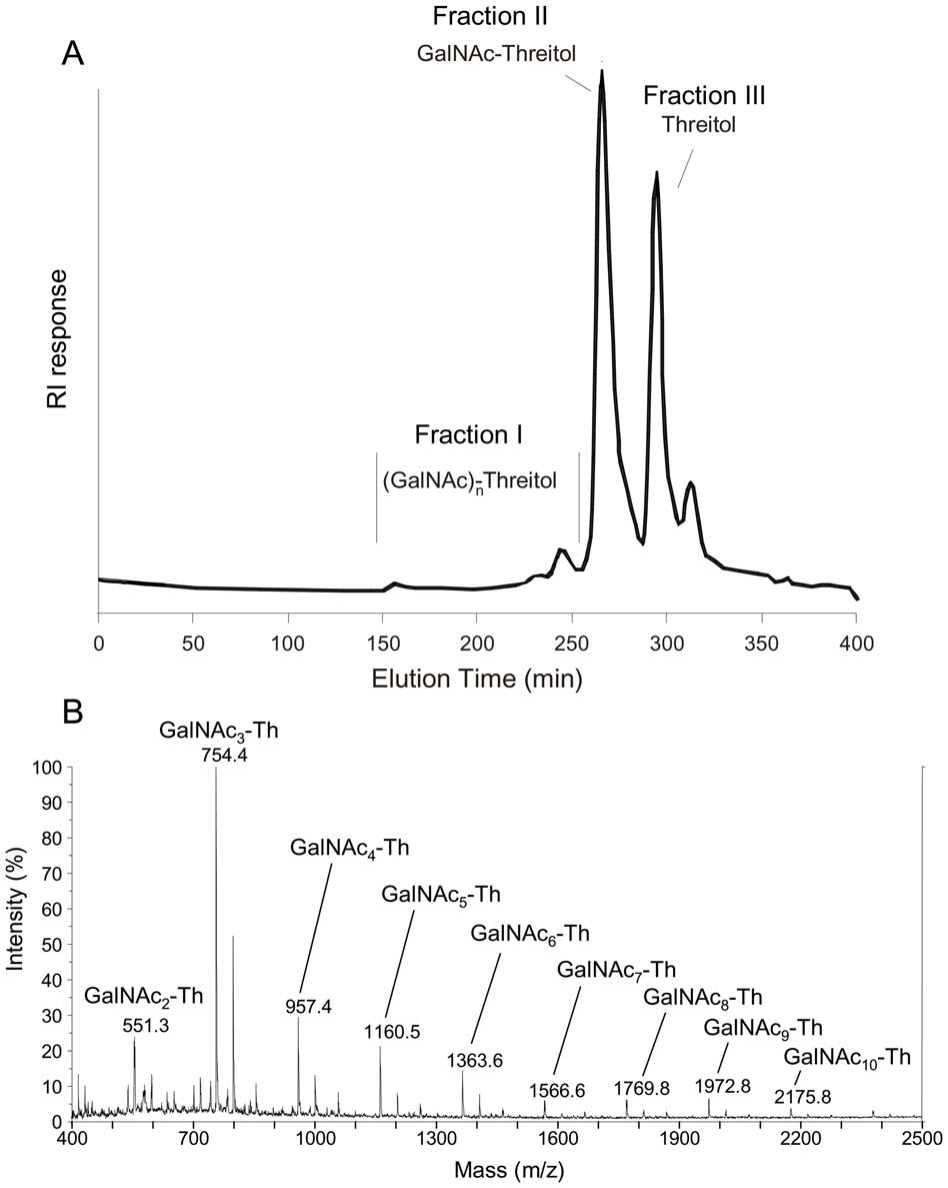
Analysis of periodate-oxidized galactosaminogalactan of *A. fumigatus*. A, Gel permeation chromatography pattern of solubilised products on a HW40S column eluted with a 0.25% acetic acid solution. The three carbohydrate containing fractions (I-III) were identified by the refractometry index (RI). B, Composition of the oligosaccharides of fraction I purified on the HW40S gel filtration; composition was based on MALDI-TOF mass spectra (mass m/z  =  [M+Na]^+^); Th: threitol (from Galactose degradation); GalNAc: N-acetylgalactosamine.

The periodate oxidation followed by mild acid hydrolysis solubilised 90% of the SGG. The insoluble product was composed of only N-acetylgalactosamine (GalNAc) residues. Three solubilised fractions were separated by gel filtration on HW40S column (Fig. 2A). GC-MS analyses showed that fraction III corresponded to threitol, resulting from the periodate degradation of 4-O-subsituted galactose residues (not shown). GC-MS analyses of permethylated fraction II showed the presence of compound with a pseudomolecular ion mass [M + H]^+^ of m/z =  424 and [M + NH_4_]^+^ of m/z  =  441 corresponding to the permethylated GalNAc-threitol (Fig. S4). NMR data confirmed this analysis and showed that the fraction II contained a compound with an α-GalNAc1-2-threitol arrangement (Table S1). MALDI-TOF analysis of compounds of fraction I indicated a mixture of GalNAc oligosaccharides linked to one threitol residue (Fig. 2B).

**Figure 3:**
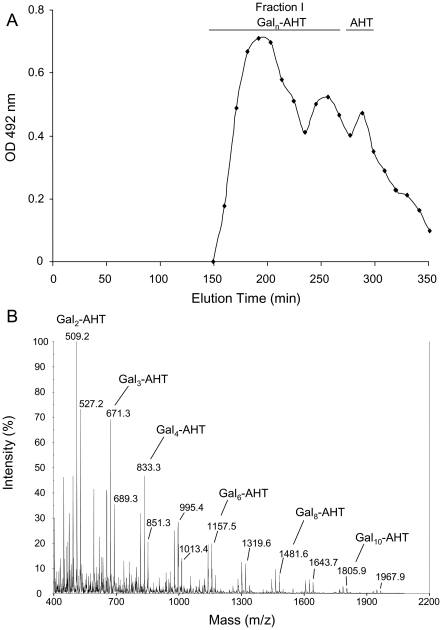
Analysis of de-N-deacetylated and nitrous deaminated galactosaminogalactan of *A. fumigatus*. A, Gel permeation chromatography pattern of solubilised products on a HW40S column eluted with a 0.25% acetic acid solution. The carbohydrate containing fractions were detected by the colorimetric phenol-sulphuric acid method. B, Composition of the oligosaccharides of fraction I purified on the HW40S gel filtration. The composition was based on MALDI-TOF mass spectra (mass m/z  =  [M+Na]^+^); AHT: 2,5-anhydrotalose (from GalNAc degradation); Gal: Galactose.

Nitrous deamination solubilised 95% of the SGG. Here, only a polygalactan that accounted for 5% of the total polysaccharide was not solubilised. The soluble material was separated on a HW40S gel permeation column. In addition to the anhydrotalose (resulting from the degradation of the galactosamine), a wide peak (fraction I) was eluted from the column (Fig.3). The MALDI-TOF analysis of fraction I revealed the presence of several pseudomolecular ion masses with a regular increase of m/z = 162 and a shift of 18, corresponding to hexose oligosaccharide linked to a non-reduced anhydrotalose in its aldehyde and hydrated forms, respectively [14] (Fig. 3). This result showed that the fraction I was composed of a mixture of galactooligosaccharides of dp 2 to 11 with an anhydrotalose at the reducing end. This result was confirmed by the NMR analysis that indicated the presence of the linkage -4-αGal1-4AHT in this fraction (Table S2).

Carbohydrate structure analyses showed that the galactosaminogalactan from *A. fumigatus* is a linear heterogeneous polymer of α1-4galactosyl and α1-4N-acetylgalactosaminyl residues. Both SGG and PGG were analyzed and showed similar structures (Table 1). The major differences between these two fractions relied on the degree of polymerization of the galactooligosaccharides and the presence of a higher amount of GalNAc in PGG. The insoluble material after periodate treatment accounted for 25% of the initial material of the PGG indicating that the homogenous linear polyN-acetylgalactosamine was 2 to 3 times higher in PGG. In addition, in contrast to SGG where galactose oligosaccharides of 2 to 10 residues were joined by one GalNAc residue, in PGG GalNAc or polyGalNAc oligosaccharides were mainly joined by a single galactose residue (Fig. S5). These data showed that the galactosaminogalactan of *A. fumigatus* did not contain a repeat unit and displayed a high heterogeneity in the sequences of oligosaccharides composed of Gal and GalNAc and that this heterogeneity impacted on the physicochemical properties of the polysaccharide.

**Table 1:** Percentage of oligosaccharide sequences found in the galactosaminogalactan of *A. fumigatus*.

	SGG	PGG
Ratio Galactose/GalNAc	60/40	15/85
Oligosaccharide sequences	Relative percentage^1^	
αGalNAc1-[4αGalNAc1-]_n_ n>10	10	25
αGal1-[4αGal1-]_n_ n>10	5	2
αGal1-[4αGalNAc1-]_2-10_-αGal1-	10	40
αGalNAc1-[4αGal1-]_2-10_αGal1-	30	10
αGal1-[4αGalNAc1-4αGal1]_n_-4αGalNAc1-	45	23

1 Percentage was estimated from the monosaccharide composition and analysis of chemically degraded products.

### Humans carry antibodies directed against GG

The antigenicity of the GG was tested first with sera from a blood bank. Surprisingly, antibodies directed against GG were present in most human sera tested: in our experimental conditions, 40% of the 131 tested sera gave by direct ELISA an OD reading >1 at a 1∶500 dilution (Fig. S6). The isotype responsible was mainly IgG2 and full inhibition of the antigen-antibody reaction was obtained with SGG, confirming the specificity of the antibody reaction. Infection with *Aspergillus* was not associated with an increase in the serum titers against GG. In a similar ELISA format with sera from aspergillosis patients, only 40% of aspergilloma patient gave an OD value higher than 1 by direct ELISA, whereas all these aspergilloma patients had high titers against the galactomannan that is a marker polysaccharide antigen of *A. fumigatus*. Similarly, only 30% of patient with invasive aspergillosis reacted positively with the galactosaminogalactan (not shown).

The lack of correlation between aspergillosis and the occurrence of high serum titers against GG in healthy patients suggested that the antibody reaction against GG was due to a cross-reactivity with α-GalNAc-containing molecules, since GalNAc has been recognised to be an immunologically reactive hexosamine present in several human or microbial antigens. Among all the molecules tested, the Tn-antigen (α-GalNAc-serine/threonine) or the serotype A marker (α-GalNAc1-3[β-Fuc1-6]β-Gal1-) did not cross react with GG (data not shown). The lack of cross reactivity with human molecules that contained a single GalNAc molecule at their non-reducing end suggested that the presence of several GalNAc molecules was required to form the immunogenic epitope. Accordingly, a high cross reactivity was found between the GG of *A. fumigatus* and the N-glycan of cell surface glycoproteins of *Campylobacter jejuni* (AcraA) that is an α1-4 linked GalNAc rich structureα-GalNAc1-4 α-GalNAc1-4 [β-Glc1-3]α-GalNAc1-4 α-GalNAc1-4 α-GalNAc1-3 β-Bac1-Asn(where Bac is 2,4-diacetamido-2,3,6-trideoxy-D-glucose; Asn, asparagine and Glc, glucose, [15]).

A significant positive correlation was calculated for the OD values obtained with the GG and AcraA molecules in 131 blood bank sera. The Spearman's rho correlation coefficient had a value of 0.71 (p<0.0001) (Fig. S6). In addition, ELISA showed that a specific rabbit polyclonal antiserum directed against the N-glycan of surface proteins of *C. jejuni* reacted positively with the GG of *A. fumigatus* (not shown). ELISA-inhibition assays were performed on a group of 30 sera with OD >1 for both AcraA and GG antigens. AcraA positive sera with OD>1 were always highly inhibited with 5 µg/ml of SGG (not shown), suggesting that the epitope recognised by these AcraA (and GG) positive sera was a linear α1-4GalNAc oligosaccharide. This result was confirmed by ELISA inhibition studies using the fraction containing exclusively the α1-4GalNAc oligosaccharide obtained after periodate oxidation or acid hydrolysis of SGG (Fig. S7). However, in 20% of serum samples, oligoGalNAc did not completely inhibit the GG recognition, indicating that, in human sera, some IgG could be specifically directed against Gal-GalNAc or Gal-Gal sequences (Fig. S7).

### GG is immunosuppressive and favors aspergillosis

**Figure 4:**
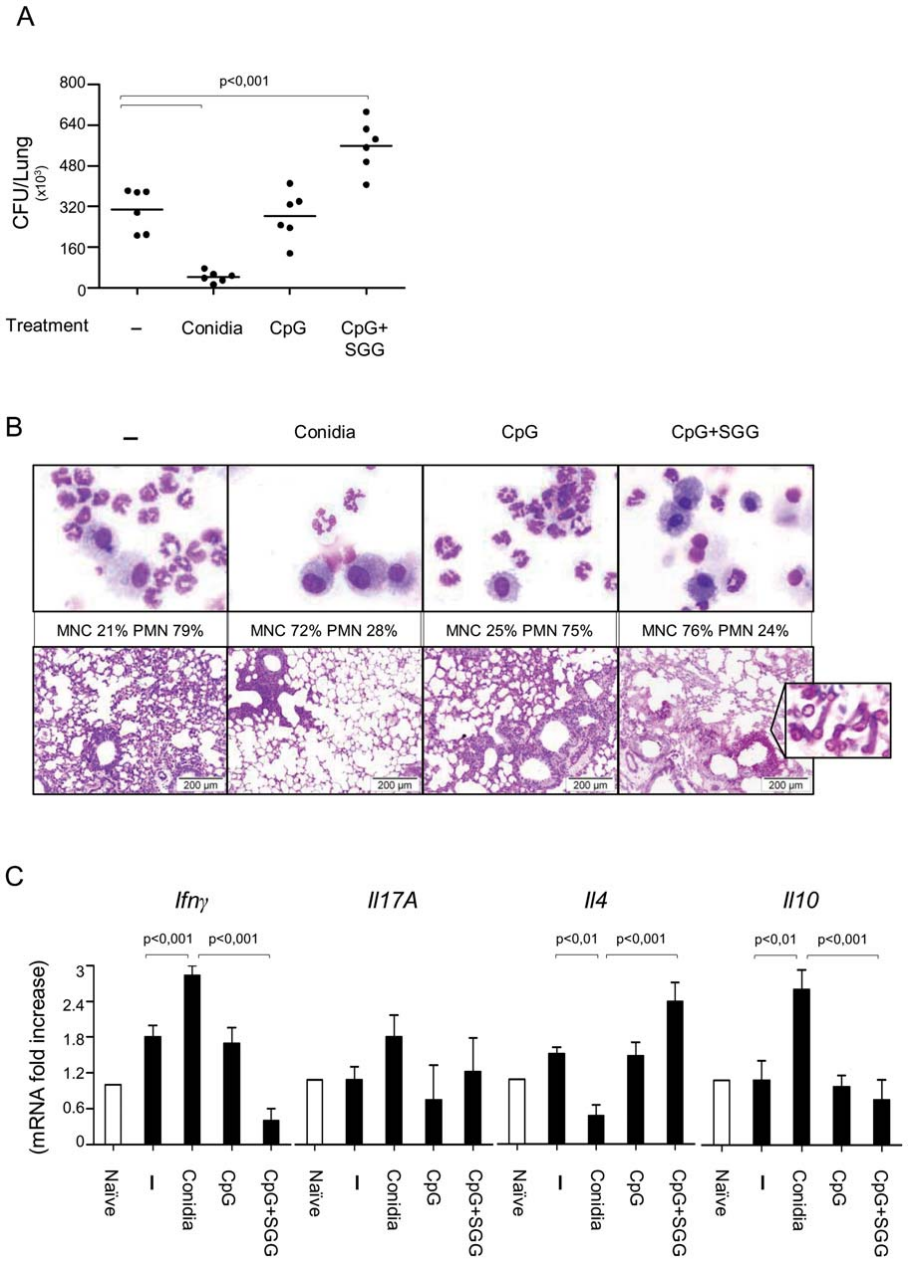
Vaccine potential of the urea soluble galactosaminogalactan (SGG) of *A. fumigatus* against invasive pulmonary aspergillosis. A, C57BL/6 mice were injected with 2×10^7^ *Aspergillus* conidia 14 days or with CpG (10 nM) or CpG and SGG (5 µg) (CpG+SGG) 14, 7 and 3 days before the intranasal infection with 2 × 10^7^ live resting conidia. Naïve are uninfected mice and – are infected, untreated mice. Fungal growth is expressed as CFUs per lung and statistical significance is indicated by a p value <0.001. B, Bronchoalveolar cells were obtained by lung lavage and lung histology (PAS-staining) was done 3 days after infection. Note that SGG failed to ameliorate inflammatory pathology and even favoured fungal growth (insert) in the absence of neutrophil recruitment. C, Cytokines were determined by RT-PCR in lung homogenates 3 days after the infection. Results pooled from 2 experiments (6 animals/group). Photographs were taken using a high-resolution Microscopy Color Camera AxioCam. Bars indicate SEM and statistical significance is indicated by p values.

Mice were treated with antigen and CpG (oligonucleotide containing umethylated CpG motifs) as adjuvant to assess the putative protective effect of this antigen against pulmonary aspergillosis in a murine model of vaccine-induced resistance [4]. Figure 4 shows that, in contrast to the protection afforded by conidia, SGG failed to confer resistance to infection and even favored fungal growth (Fig. 4A). No reduced inflammatory pathology was seen in the lung where actual fungal growth was observed in GpG+SGG-treated mice (Fig. 4B) and the cytokine pattern showed that SGG inhibited *Ifn*γ*/Il10* and activated *Il4* gene expression in the TLN, thus suggesting inhibition of protective Th1/Treg cells and promotion of Th2 responses (Fig. 4C).

**Figure 5:**
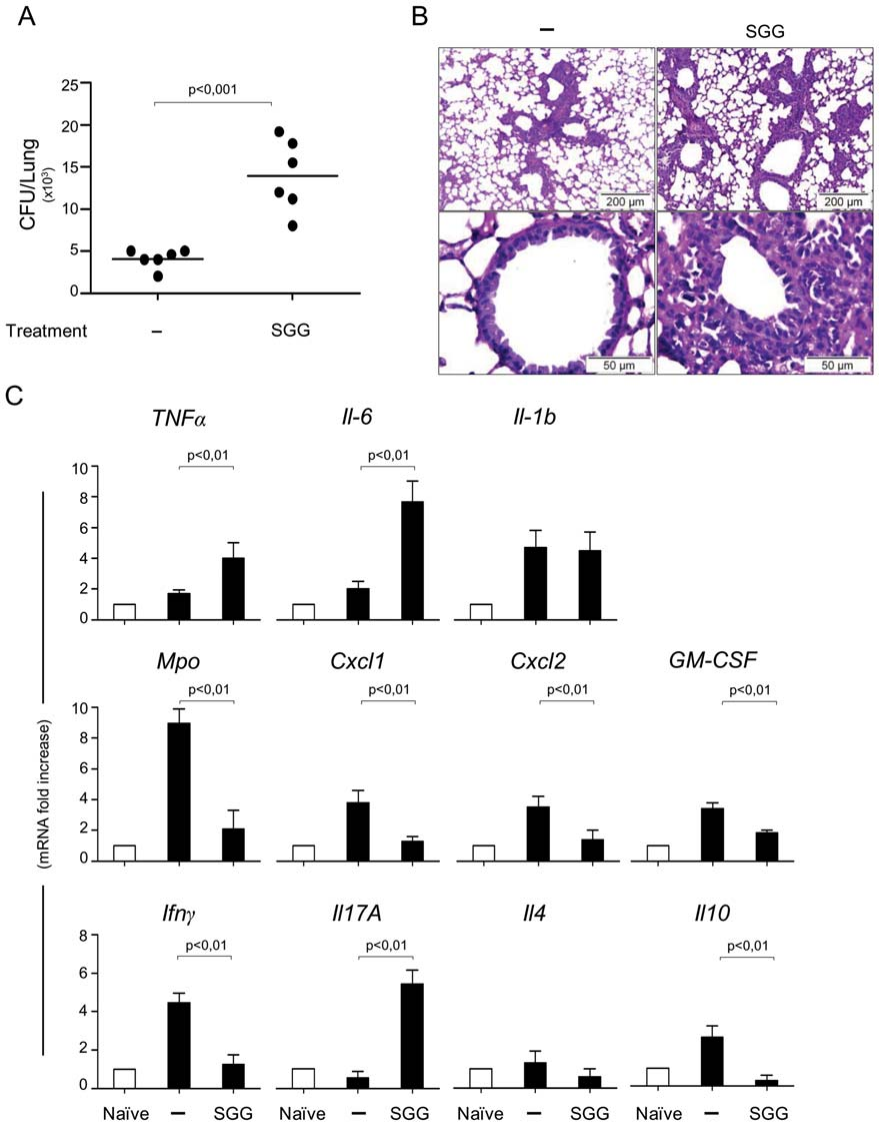
Impact of SGG on primary aspergillosis in intact mice. A, C57BL/6 mice were first injected with SGG at day 3, 2 and 1 before conidial inhalation and infected on day 0 with 2×10^7^ *Aspergillus* conidia. Naïve are uninfected mice, – are infected, untreated mice and SGG are mice that have received SGG (250 mg/kg i.n. the day of the infection and on days 1, 2 and 3 post-infection). Fungal growth is expressed as CFUs per lung and statistical significance is indicated by a p value <0.001. Results pooled from 2 experiments (6 animals/group) with one example shown in panel A. B, Lung histology (PAS-staining) of mice treated as indicated, 3 days after infection showing signs of inflammatory pathology in the immunocompetent mice treated with SGG. C, Cytokines were determined by RT-PCR in lung homogenates 3 days after the infection. Note that SGG induced inflammatory cytokine gene expression, such as *Tnfα*, *Il6*, *Il17a* and *Il4* genes, but suppressed *Ifnγ* and *Il10* expression; the low level of *Mpo* gene expression is in agreement with the low counts of neutrophils in the lung of infected mice. Bars indicate SEM and statistical significance is indicated by p values.

Most interestingly, when the immunomodulatory activity of SGG was assessed in intact mice with primary infection, SGG promoted the infection, as seen by the increased lung CFUs and inflammatory pathology in SGG-treated mice as compared to controls (Fig.5A and B). Figure 5C shows that SGG induced inflammatory cytokine gene expression, such as *Tnfα* and *Il6*. Moreover, SGG induced the expression of *Il17a* genes but suppressed *Ifnγ* and *Il10* expression. These data were in agreement with the expression of the relative Th cell specific transcription factors in the TLN (data not shown). Of interest, SGG appeared to reduce neutrophil infiltrates in the lung during infection, as also seen by the reduced *Mpo* expression (Fig. 5C). These data suggest that SGG inhibit host defence against *A. fumigatus.*

### GG induces neutrophil apoptosis

**Figure 6:**
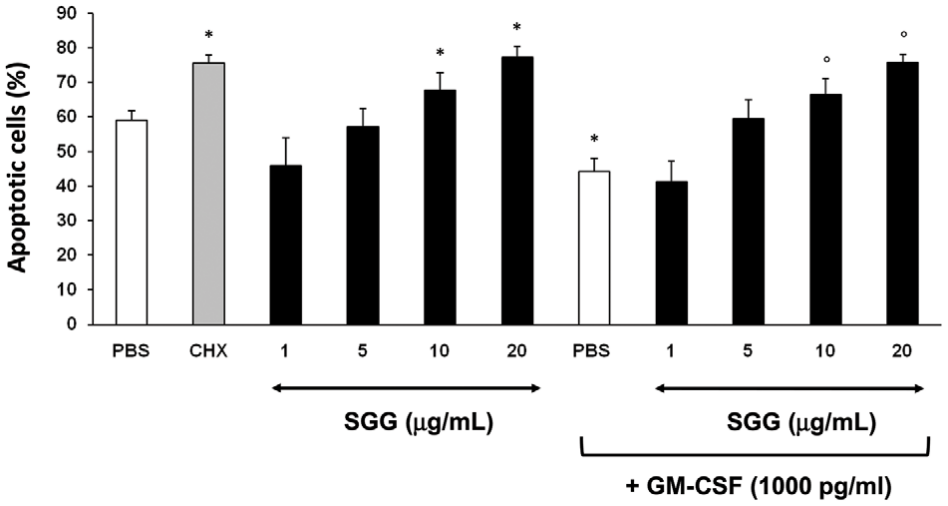
SGG induces neutrophil apoptosis. Whole-blood samples (500 µl) were incubated in 24-well tissue culture plates at 37°C for 20 h with 5% CO_2_ with PBS or SGG (1–20 µg/ml). Cycloheximide (CHX) (10 µg/ml) and GM-CSF (1000 pg/ml) were used as proapoptotic and antiapoptotic controls, respectively. Samples (100 µl) were incubated with APC-conjugated anti-CD15 and stained with FITC-conjugated annexin V and 7-AAD as described in Materials and Methods. Results are expressed as the percentage of total apoptotic cells (early and late apoptotic cells). Values are means ± SEM (n = 4). *Significantly different from sample incubated with PBS (p< 0.05). ° Significantly different from sample incubated with GM-CSF (p<0.05).

Bloodstream neutrophils have a short half-life and prolongation of their lifespan is critical for efficient pathogen destruction. As SGG-treated mice exhibited reduced neutrophil infiltrates in the lung during infection as compared to controls, we investigated the effect of SGG on neutrophil apoptosis. Neutrophils cultured at 37°C died rapidly by apoptosis, about 60% of cells being annexin V^+^ after 20 h. As previously reported [16], apoptosis was accelerated by cycloheximide and delayed by GM-CSF. The percentage of apoptotic cells in whole-blood samples incubated with SGG (10–20 µg/ml) was significantly higher than in the PBS control. In addition, SGG significantly inhibited GM-CSF-induced PMN survival (Fig. 6).

### Macrophage galactose-type lectin (MGL) and GG

**Figure 7:**
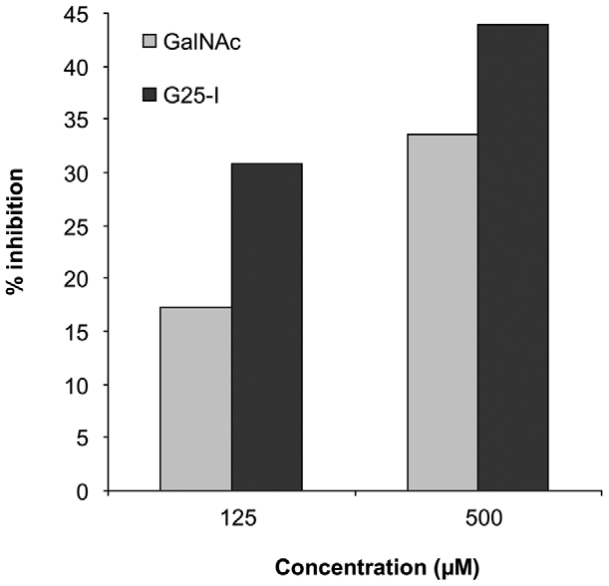
Binding of macrophage galactose-type lectin (MGL) to the galactosaminogalactan of *A. fumigatus*. ELISA-inhibition of MGL-Fc interacting with α-GalNAc-conjugated polyacrylamide (GalNAc-PAA) by free GalNAc monosaccharides and a mixture of oligosaccharides exclusively composed of N-acetylgalactosamine with an average degree of polymerisation of 7.5 (G25-I). ELISA plates were coated with GalNAc-PAA (2 µg/ml). Plates were blocked with 1% BSA and the recombinant MGL-Fc chimera was added (0.5 µg/ml) for 2 h at room temperature in the presence of 0.125 and 0.25 mM of SGG hydrolysate fractions (G25-I). Binding was detected using a peroxidase-labeled anti-human IgG-Fc antibody.

Since the C-type lectin MGL has been shown to be specific for GalNAc residues, the binding of GG to MGL was investigated. Using SGG and PGG as ligands, ELISA experiment showed a lack of specific interaction of the GG of *A. fumigatus* and recombinant MGL-Fc. Similarly, immunofluorescence experiments showed that MGL-Fc did not bind to the cell wall of germinated conidia expressing GG on their surface (data not shown). ELISA inhibition using GalNAc coupled to polyacrylamide (GalNAc-PAA) as the ligand showed that GG did not inhibit the binding of GalNAc-PAA to MGL. In contrast, GalNAc oligosaccharides obtained by HCl hydrolysis (Fig. S8) inhibited the interaction with MGL (Fig. 7). Since MGL recognized terminal GalNAc residues and since the average degree of polymerisation of the oligosaccharide fraction used was 7.5, the relative inhibition was similar for GalNAc and the GalNAc oligosaccharide pool when expressed in molar concentration. In contrast to GalNAc monomers that inhibit 100% of the binding at 1 mg/ml in our experimental conditions, no full inhibition was obtained with the oligoGalNAc fraction because at concentrations higher than 500 µg/ml, the GalNAc oligosaccharides precipitated. The lack of binding of MGL to the whole GG was due to the presence of one terminal GalNAc per 700 GalNAc residues in average in the linear 100 kDa GG polysaccharide. Only oligoGalNAc resulting from the degradation of GG can be recognised efficiently by MGL.

## Discussion

Here, we describe the purification and the chemical characterization of a new galactosaminogalactan secreted by the mycelium of *A. fumigatus*. Cell wall and extracellular polysaccharides containing galactosamine residues have been also identified in other filamentous fungi, such as *Neurospora, Rhizopus, Helminthosporium, Penicillium and Aspergillus* species [17], [18]. However, the structure of these polysaccharides has been poorly characterized with linkages that can be either α1-4 and/or α1-3 linkages with part of the GalNAc molecules being N-deacetylated [19], [20], [21], [22]. The *A. fumigatus* galactosaminogalactan is exclusively composed of α1-4linked galactose and α1-4linked N-acetylgalactosamine residues. In our growth conditions, the GG was totally N-acetylated. It was, however, shown that this linear polysaccharide is extremely heterogeneous, with strands of galactose and N-acetylgalactosamine of variable length that impact on the polysaccharide solubility and putatively on biological properties. This heterogeneity is unique to the galactosaminogalactan because the other constitutive cell wall polysaccharides of *A. fumigatus* are homopolymers (chitin, glucans) or have well defined repeating unit, such as *A. fumigatus* galactomannan [12], [23]. The main motif is Gal-GalNAc, but the variable Gal/GalNAc ratio inside each polymer chain suggests random synthesis of the polymer, as in some plant polysacharides [24]. The synthesis of polygalactose and polyN-acetylgalactosamine oligosaccharides, as well as the synthesis of repetitive Gal-GalNAc unit is totally unknown. The galactose of GG is present in a pyranose form, whereas the galactose of the galactomannan, which is a major antigen of *A. fumigatus*, is in a galactofuranose form. *A. fumigatus* has the ability to synthesise the two isoforms of galactose, like many bacterial, parasite and fungal microorganisms [25], [26], [27], [28], [29]. This was indeed shown in a UDP-Gal epimerase mutant, in which galactofuranose synthesis was abolished, but some galactose was still present in the cell wall, corresponding to the GG [30] (data not shown.).

It was very surprising to see that a majority of the sera from the blood bank had high titers of IgG against GG, with GalNAc residues being the main determinant for the antigenicity. This result suggested that this polysaccharide could be a very potent immunoadjuvant that could be used to induce the production of antibodies against poorly antigenic molecules. The only cross-reacting antigen identified so far was the N-glycans of glycoproteins of *C. jejuni*. This result suggests that the portal of entry for the GG could be the gut barrier as has been demonstrated for the galactomannan polymer [31], [32]. N-glycoproteins of *C. jejuni* can bind to human intestinal epithelial cells [33], [34]; Gal/GalNAc rich-polysaccharides are produced by many environmental fungal food contaminants including *Aspergillus* and *Penicillium* species suggesting that in both cases α1-4GalNAc oligosaccharides could cross the intestinal epithelium.

This galactosaminogalactan study has confirmed the essential immunological role of the fungal cell wall polysaccharides. This has been seen with all medically important fungi [6], [35], [36], [37]. Some of the cell wall polysaccharides of *A. fumigatus*, such as α1-3glucan and β1-3glucan chains, have been shown to induce a protective immune response through the activation of Th1, Th17 or Treg responses and the inhibition of the Th2 response [4]. A different immunological function can be conveyed by other cell wall polysaccharides. GG not only is not inducing a protective response but is promoting an immunosuppressive function that can trigger disease in immunocompetent mice. A similar function can probably be attributed to the galactomannan that also has been shown to induce a Th2/Th17 response that was not protective [4]. However, at that time the authors did not investigate the immunosuppressive role of the later polysaccharide in immunocompetent mice. The production of a Th2/Th17 response is in agreement with the presence of anti-GG and anti-Galactomannan IgG2 antibodies in human sera, whereas the level of anti-α1-3glucan and β1-3glucan antibodies in humans is absent or extremely low. In addition, GG-induced PMN death may be involved, at least in part, in the decrease in neutrophil infiltrates in lungs from GG-treated mice despite an increased Th17 response. The GG is the first *Aspergillus* polysaccharide that induces cell apoptosis. The pathogenic yeast, *Cryptococcus neoformans* produces a polysaccharide capsule constituted by 2 polymers: glucuronoxylomannan and galactoxylomannan that induce *in vitro* apoptosis of human macrophages and T-cells [38], [39].

Polysaccharide receptors of mammalian macrophages remain poorly characterized. Besides Dectin1 that recognizes β1-3glucans, receptors able to recognize α1-3glucans or galactan have not been identified yet [40], [41]. The MGL (macrophage galactose-type lectin) was the obvious candidate for GG binding since it recognizes specifically GalNAc residues [42]. This receptor is located at the cell surface of immature dentritic cells and has been shown to be involved in the recognition of pathogens through GalNAc residues and in the retention of immature DCs in peripheral tissue and lymphoid organs [42], [43], [44]. The MGL is able to bind to N-glycoproteins of *C. jejuni* through α1-4 linked GalNAc residues [45] and the binding of these N-glycans to MGL influences the function of human dentritic cells. However, no specific binding of GG to human MGL-Fc was seen, suggesting that cell surface MGL was not involved in the recognition of *A. fumigatus* GG. However, the intracellular hydrolysis of GG, as shown for some bacterial polysaccharides [46], may release oligosaccharides that can bind to MGL that has been seen in endocytic compartments. Such intracellular recognition of GG oligosaccharides could then induce the pro-inflammatory response. This hypothesis is currently being investigated.

## Materials and Methods

### Strain, media and galactosaminogalactan production

The *A. fumigatus,* strain CBS 144–89 was grown in a 15l fermenter in modified Brian medium (2% asparagine, 5% glucose, 2.4 g/l NH_4_NO_3_, 10 g/l KH_2_PO_4_, 2 g/l MgSO_4_-7H_2_O, 26 mg/l ZnSO_4_-7H_2_O, 2.6 mg/l CuSO4-5H_2_O, 1.3 mg/l Co(NO_3_)_2_-6H_2_O, 65 mg/l CaCl_2_, pH 5.4) for 72 h at 25°C. The mycelium was removed by filtration under vacuum and the supernatant was precipitated with 2.5 vol. of ethanol overnight at 4°C. The pellet was collected by centrifugation (3000g, 10 min). The pellet was washed twice with 2.5 l of 150 mM NaCl and then extracted with 8 M urea (2 h twice at room temperature under shaking). Urea-supernatants (SGG) were pooled and extensively dialyzed against water and freeze-dried. Urea-insoluble pellet (PGG) was washed with water and freeze-dried.

### Monosaccharide analysis

Total hexoses were measured by the phenol-H_2_SO_4_ method using galactose as a standard [47]. Total hexosamines were determined with *p*-(dimethylamino)-benzaldehyde reagent after 4 h of 8N HCl hydrolysis at 100°C using galactosamine as a standard [48]. Monosaccharides were identified by GC as their alditol acetates after total acid hydrolysis (trifluoroacetic acid (TFA) 4N or HCl 4N, 100°C, 4 h) [49]. Threitol resulting from the periodate oxidation of galactose was identified by GC-MS and NMR. In absence of reference spectrum, anhydrotalose resulting from the nitrous deamination of galactosamine was identified by GC-MS by comparison with the mass spectrum of anhydromannitol and by NMR.

### Methylation

Prior to the methylation procedure, polysaccharides were peracetylated as previously described [50]. Dried sample (2 mg) was methylated by the DMSO/lithium methyl sulfinyl carbanion/ICH_3_ procedure [50]. After hydrolysis of the permethylated sample (4 N TFA 100°C, 4 h), borodeuteride-reduction and peracetylation, methyl ethers were identified by GC-MS. Oligosaccharides were permethylated by the DMSO/NaOH/ICH_3_ procedure [51].

### Periodate oxidation

Polysaccharide fractions (30 mg) were resuspended in 4 ml of 10 mM HCl at 50°C for 24 h and then oxidized with 100 mM sodium *m*-periodate at 4°C in darkness during 7 days. Excess reagent was destroyed by adding 0.5 ml of ethylene glycol. The solution was dialysed against water and freeze-dried. The material was reduced overnight by 10 mg/ml NaBH_4_ at room temperature. After neutralisation to destroy the excess of borohydride, reduced oxidized polysaccharide was dialysed against water and freeze-dried. A mild acid hydrolysis was performed by 1.5 ml of 50 mM TFA at 100°C for 1 h. The solubilised fraction was fractionated on a HW40S column (TosoHaas, 90×1.4 cm) equilibrated in 0.25% acetic acid at the flow rate of 0.4 ml/min. Eluted sample were detected by refractometry. The insoluble fraction was washed twice in water.

### Nitrous acid deamination

Polysaccharide fractions (30 mg) were resuspended in 4 ml of 10 mM HCl at 50°C for 24 h and then de-N-acetylated with 40% NaOH (final concentration) at 100°C for 4 h. After neutralisation by addition of acetic acid, samples were dialysed and freeze-dried. Dried samples were resuspended in 600 µl of NaOAc 0.5 M pH 4. The deamination was started by addition of 300 µl of 1 M NaNO_3_ and performed at 50°C during 3 h with the addition of 300 µl of 1 M NaNO_2_ each hour. Soluble degraded products were fractionated by gel filtration chromatography through a HW40S column, as described above. Neutral sugars were detected by the phenol-H_2_SO_4_ method [47].

### Partial acid hydrolysis

10 mg of polysaccharide were treated with 1 ml of 0.1 M HCl for 3 h at 100°C. After neutralisation with 1% Na_2_CO_2,_ solubilised materials were purified by gel filtration through a Sephadex G25 column (GE Heathcare, 90×1.4 cm) and eluted with 0.25% acetic acid at a flow rate of 9 ml/h.

### Molecular weight analysis by gel filtration chromatography

Due to its insolubility, carboxymethylation of GG was necessary to estimate its molecular size by gel filtration. The polysaccharide (0.2 g) was carboxymethylated by addition of 20 ml of 1.6 M NaOH and 0.3 g of monochloroacetic acid. The mixture was heated at 75°C and stirred magnetically for 8 h. After neutralisation, the solution was dialysed against water and freeze-dried. The carboxymethylated polysaccharide was soluble in 0.5% acetic acid and 10 mg were deposited onto a Sephacryl S400 column (Pharmacia, 90×1.4 cm) at the flow rate of 10 ml/h. Dextrans (Pharmacia, T2000, T500, T70, T40) were used as standards for the column calibration.

### GC and GC-MS

GC was performed on a Perichrom PR2100 instrument with a flame ionisation detector using a capillary column (30 m×0.32 mm id) filled with a DB-1 (SGE) under the following conditions: gas vector and pressure, helium 0.7 bar; temperature program 120 to 180°C at 2°C/min and 180 to 240°C at 4°C/min. GC-MS was performed on an EI/CI mass spectrometer detector (model 5975C, Agilent technologies, Massy France) coupled to a chromatograph (model 7890A), using a HP-5MS capillary column (30 m×0.25 mm id, Agilent technologies) under the following conditions: gas vector: helium at 1.2 ml/min; temperature program: 100 to 240°C at 8°C/min and 240°C for 10 min. Ammoniac gas was used for the chemical ionisation.

### Matrix-assisted desorption ionisation/Time of flight (MALDI-TOF) mass spectrometry

MALDI-TOF mass spectra were acquired on a Voyager Elite DE-STR mass spectrometer (Perspective Biosystems, Framingham, MA, USA) equipped with a pulsed nitrogen laser (337 nm) and a gridless delayed extraction ion source. The spectrometer was operated in positive reflectron mode by delayed extraction with an accelerating voltage of 20 kV and a pulse delay time of 200 ns and a grid voltage of 66%. Samples were prepared by mixing directly on the target 0.5 µl of oligosaccharide solution in water (10–50 pmol) with 0.5 µl of 2,5-dihydroxybenzoic acid matrix solution (10 mg/ml in CH_3_OH/H_2_O, 50∶50, V/V). The samples were dried for about 5 min at room temperature. Between 50 and 100 scans were averaged for every spectrum.

### NMR Spectroscopy

NMR spectra of the polysaccharides were acquired at 318 and/or 343 K on a Varian Inova 500 spectrometer equipped with a triple resonance ^1^H{^13^C/^15^N} PFG (pulsed field gradient) probe whereas spectra of either nitrous deamination or periodate oxidation products were acquired at 298 K on Varian Inova 500 and 600 spectrometers equipped with a triple resonance ^1^H{^13^C/^15^N} PFG and a cryogenically-cooled triple resonance ^1^H{^13^C/^15^N} PFG probe respectively (Agilent technologies, Massy France). Polysaccharidic samples solubilized in acetic acid 0.05%V/V in H_2_O by warming for one hour at 100°C were freeze dried and redissolved in DCl 0.06 M in D_2_O (DCl ≥ 99.0% ^2^H atoms and D_2_O ≥99.9% ^2^H atoms, Euriso-top, Saint-Aubin, France). After a second freeze-drying, they were redissolved in D_2_O and transferred in a 5 mm NMR tube (Wilmad 535-PP, Interchim, Montluçon, France). The final concentration was about 5 mg/mL. Samples were dissolved in D_2_O and transferred in a 5 mm NMR tube (Shigemi BMS-005 V, Shigemi Inc., Alison Park, United States). ^1^H chemical shift were referenced to external DSS (2,2-methyl-2-silapentane-5-sulfonate sodium salt hydrate, its methyl resonance was set to 0 ppm). ^13^C chemical shifts were then calculated from ^1^H chemical shift and gamma ratio relative to DSS. ^13^C/^1^H gamma ratio of 0.251449530 was used [52].

The following strategy was used for assignment of nuclei. First, the non-exchangeable proton resonances of intra glycosidic residue spin systems were assigned using two-dimensional COSY (correlation spectroscopy), relayed COSY (up to two relays) and TOCSY (Total correlation spectroscopy; with mixing time ranging from 30 to 120 ms) experiments [53]. Secondly, ^1^H-^13^C edited gHSQC (Gradient selected heteronuclear single-quantum correlation) and gHSQC-TOCSY (mixing time up to 80 ms) experiments allowed the ^13^C chemical shifts assignment from previously identified ^1^H resonances [54]. Then, ^1^H,^1^H coupling constants for the oligosaccharides were extracted from 1D and/or 2D spectra (^1^H resolution of 0.1 Hz and 0.6 Hz respectively) and the anomeric configuration was established from the knowledge of ^3^J_1,2_ value. Finally, the interglycosidic linkages determination was achieved with ^1^H-^1^H NOESY (Nuclear overhauser effect spectroscopy) experiments for the polysaccharides (mixing time of 15 and 50 ms) and with ^1^H-^1^H ROESY experiments (mixing time of 250 ms) [55] and/or ^1^H-^13^C gHMBC (Gradient selected heteronuclear multiple bond correlation) experiment (long range delay of 60 ms) [54] for the oligosaccharides.

### Ethics statement

Patient samples were collected according to French Ethical rules. Written informed consent and approval by institutional review Board at the Pitié-Salpêtrière Hospital, at the Etablissement français du sang and at Saint-Louis Hospital were obtained.

Mouse experiments were performed according to the Italian Approved Animal Welfare Assurance A–3143–01. Legislative decree 157/2008-B regarding the animal licence was obtained by the Italian Ministry of Health lasting for three years (2008–2011). Infections were performed under avertin anesthesia and all efforts were made to minimize suffering.

### Analysis of anti-galactosaminogalactan antibodies

Serum samples from 131 healthy subjects (from Groupe français du sang and Hôpital Saint-Louis, Paris), 25 invasive aspergillosis patients (Hôpital Saint-Louis; kind gift of A. Sulhaian) and 5 aspergilloma patients (CHU Toulouse; kind gift of P. Recco) were used through this study. Blood group was determined by the Etablissement français du sang. The presence of antibodies directed against the *A. fumigatus* galactosaminogalactan was assessed by a direct enzyme-linked immunosorbent assay method (ELISA). Purified *A. fumigatus* galactosaminogalactan and AcraA, a recombinant N-glycoprotein from *Campylobacter jejuni* expressed in *E. coli* [56], [57] were used as antigens. Wells of microdilution plates (F-form, Greiner, Frickenhausen, Germany) were coated with 100 µl of a suspension of 1 µg/ml galactosaminogalactan (PGG) or 5 µg/ml AcraA diluted in 50 mM Na_2_CO_3_ pH 9 and incubated overnight at room temperature. Binding of antibodies to the ELISA-plate was estimated with patient sera diluted 1∶500 and peroxidase-conjugated anti-human immunoglobulin G, as previously described [12]. Cross reactivity between GG and the Tn antigen (α-GalNAc-Serine) was analysed by ELISA with a monoclonal antibody against the Tn antigen (kind gift from Dr R. Lo-Man, Institut Pasteur).

### Production of anti-galactosaminogalactan monoclonal antibody

Mice (Balb-C) were immunized subcutaneously with a crude cell wall preparation of *A. fumigatus* mycelium. Monoclonal antibodies have been produced by F. Nato and P. Beguin (Plateforme technique de protéines recombinantes et anticorps monoclonaux, Institut Pasteur) as previously described [58]. Screening of positive hybridoma was followed by ELISA using the HCl-treated PGG as specific antigen. These mAbs did not react with other Aspergillus polysaccharides, such as galactomannan, β1-3glucan, α1-3glucan. ELISA-inhibition experiments showed that the recognition of mAb-galactosaminogalactan was fully inhibited by oligoGalNAc obtained after partial HCl hydrolysis and gel filtration chromatography on G25 sephadex column as described above (Fig. S8)

### Immunofluorescence

Resting conidia and conidia germinated for 8 h in a 2% glucose/1% peptone liquid medium were fixed with 2.5% *p*-formaldehyde (PFA) overnight at 4°C. After fixation, cells were washed with 0.2 M glycine in PBS for 5 min, then with 5% goat serum in PBS for 1 h. Cells were incubated with the anti-galactosaminogalactan monoclonal antibody at 20 µg Ig/ml in 5% goat serum/PBS for 1 h at room temperature. After washing with PBS-BSA 1%, cells were incubated with a goat FITC-conjugated Ab directed against mouse IgG(H+L) diluted 1∶100 in goat serum/PBS. After washing in PBS, cells were visualized with an inverted fluorescence light microscope. Specificity of labelling was assessed by preincubation of MAb with 50 µg/ml of G25-I fraction (Fig. S8).

### Binding to MGL assay

Binding assay to the macrophage galactose lectin (MGL) was done by ELISA-inhibition using a recombinant MGL-Fc chimeric protein as previously described [42]. Briefly, α-GalNAc-conjugated polyacrylamide (2 µg/ml, Lectinity) was coated on ELISA plates. Plates were blocked with 1% BSA and the MGL-Fc was added (0.5 µg/ml) for 2 h at room temperature. For inhibition assays, MGL-Fc was previously incubated for 1 h at room temperature in the presence of increasing concentrations of SGG, GalNAc oligosaccharides, Gal or GalNAc or 10 mM EGTA. Binding was quantified using a peroxidase-conjugated secondary antibody directed against human IgG Fc (Jackson).

The putative binding of MGL to mycelium was investigated by immunofluorescence. For that purpose, 0.4×10^5^ conidia were incubated in 200 µl of Brian's medium in wells of chamber slides (Lab-Tek, Nunc) at 37°C for 9 h, washed with PBS and fixed in 2.5% PFA overnight. Cells were washed with 0.2 M glycine in TSM buffer (20 mM TrisHCl; 150 mM NaCl, 2 mM MgCl_2_, 1 mM CaCl_2_, pH 7.4) for 5 min, then with 5% goat serum in TSM for 1 h. Cells were incubated with the MGL-Fc at 65 µg/ml in 5% goat serum/TSM for 1 h at room temperature. After washing with TSM, cells were incubated with an anti-human Fc FITC conjugated-goat anti-serum at 15 µg/ml in goat serum/TSM. After washing in TSM, then water, cells were visualized under a fluorescent light microscope.

### Mouse experiments

Female, 8- to 10-week-old inbred C57BL6 (*H-2^b^* ^)^ mice were obtained from Charles River Breeding Laboratories (Calco, Italy). The vaccination model was as previously described [4]. Briefly, mice were injected intranasally with 2×10^7^ *Aspergillus* conidia/20 µl saline 14 days before the infection or with 5 µg SGG + 10 nM CpG oligodeoxynucleotide 1862 (CpG)/20 µl saline, administered 14, 7 and 3 days before the intranasal infection. Mice were immunosuppressed with 150 mg/kg/i.p. of cyclophosphamide a day before infection and then intranasally infected with a suspension of 2×10^7^ viable conidia/20 µl saline. In another set of experiments, immunocompetent mice were injected with 250 mg/kg SGG i.n. the day of the infection (2×10^7^ viable conidia/20 µl saline) and on days 1, 2 and 3 post-infection. Mice were monitored for fungal growth (CFU/organ expressed as mean ± SEM) as described [59]. For histology, sections (3–4 µm) of paraffin-embedded tissues were stained with periodic acid-Schiff (PAS). Cytokines were quantified by Real-time PCR, performed using the Stratagene Mx3000P QPCR System, and SyBR Green chemistry (Stratagene Cedar Creek, Texas). Total lung cells were recovered 3 days after the infection. CD4^+^ T cells (>99% pure on FACS analysis) from thoracic lymph nodes (TLNs) recovered 7 days after the infection, were separated by magnetic cell sorting with MicroBeads and MidiMacs (Miltenyi Biotec). Cells were lysed and total RNA was extracted using RNeasy Mini Kit (Qiagen) and reverse transcribed with Sensiscript Reverse Transcriptase (Qiagen), according to manufacturer's directions. The PCR primers were as described [60], [61]. Amplification efficiencies were validated and normalized against *Gapdh*. The thermal profile for SYBR Green real-time PCR was at 95°C for 10 min, followed by 40 cycles of denaturation for 30 seconds at 95°C and an annealing/extension step of 30 seconds at 72°C. Each data point was examined for integrity by analysis of the amplification plot. The mRNA-normalized data were expressed as relative cytokine mRNA in treated cells compared with that of mock-infected cells.

### Measurement of neutrophil apoptosis

Neutrophil apoptosis was quantified by using annexin V and the impermeant nuclear dye 7-amino-actinomycin D (7-AAD) as previously described [16]. Apoptosis was measured after incubation in 24-well tissue culture plates at 37°C with PBS or SGG (1-20 µg/ml) for 20 h. Cycloheximide (Calbiochem, La Jolla, CA) (10 µg/ml) and GM-CSF (R&D Systems) (1000 pg/ml) were used as proapoptotic and antiapoptotic controls, respectively. In some experiments, blood samples were first incubated with SGG for 1 h and then with GM-CSF. Whole-blood samples (100 µl) were then washed twice in PBS, incubated with allophycocyanin (APC)-anti-CD15 mAb (BD Biosciences) for 15 min, and then incubated with fluorescein (FITC)-annexin V (BD Biosciences) for 15 min. After dilution in PBS (500 µl), the samples were incubated with 7-AAD (BD Biosciences) at room temperature for 15 min and analyzed immediately by flow cytometry (Gallios^TM^, Beckman Coulter). Neutrophils were identified as CD15^high^ cells and 2×10^5^ events were counted per sample. The combination of FITC-annexin V and 7-AAD was used to distinguish early apoptotic cells (annexin V^+^/7-AAD^-^), from late apoptotic cells (annexin V^+^/7-AAD^+^), necrotic cells (annexin V^-^/7-AAD^+^) and viable cells (unstained).

### Statistical analysis

Statistical analyses of the ELISA data were performed using the Spearman's rho test with the JMP software (SAS; Cary, NC).

Data from mouse experiments were analyzed by GraphPad Prism 4.03 program (GraphPad Software, San Diego, CA). Student's *t* test or analysis of variance (ANOVA) and Bonferroni's test were used to determine the statistical significance (*P*) of differences in organ clearance and *in vitro* assays. The data reported are either from one representative experiment out of three to five independent experiments (western blotting and RT–PCR) or pooled from three to five experiments, otherwise. The *in vivo* groups consisted of 6–8 mice/group.

Data on the measurement of neutroplil apoptosis are reported as means ± SEM. Comparisons were based on ANOVA and Tukey's Post Hoc tests, using Prism 3.0 software (GraphPad software).

## Supporting Information

Figure S1 **Gas-liquid chromatography of methyl ethers obtained after permethylation of the galactosaminogalactan of** ***A. fumigatus*** **.** Methyl ethers (2,3,6-Gal: 2,3,6-tri-O-methyl-1,4,5-tri-O-acetyl-galactitol; 3,6-GalNAc: 3,6-di-O-methyl-1,4,5-tri-O-acetyl-N-methyl-N-acetyl-galactosaminitol) were obtained after hydrolysis, reduction and acetylation of the permethylated GG.
(PPT)

Figure S2 **Gel permeation analysis of carboxymethylated urea-soluble galactosaminogalactan of** ***A. fumigatus.*** Gel permeation was performed on a Sephacryl S400 column. Dextrans (Pharmacia, T2000, T500, T70, T40) were used as standards for the column calibration. Fractions were detected by the refractometry index.
(PPT)

Figure S3 **1D ^1^H and 2D ^1^H, ^13^C HSQC spectrum of carboxymethylated galactosaminogalactan (SGG, urea-soluble GG; PGG, urea-insoluble GG).** The 1D ^1^H and 2D ^1^H, ^13^C gHSQC spectra of carboxymethylated GG fractions exhibited two main signals in the sugar anomeric region at 5.003/103.07 and 5.287/99.07 ppm compatible with α-anomers. No β-anomeric configuration was observed. The methyl signal at 2.032/24.61 ppm, characteristic of an acetyl group, and the proton at 5.287 ppm, correlated with the typical upfield shifted H_2_/C_2_ (3.587/53.68 ppm) of a 2-N-acetylation, were in agreement with the presence of N-acetylgalactosamine. NMR data showed downfield shifts for the carbone-4 of both sugar residues, indicating their 4-O substitution and their pyranose configuration that was in agreement with the NOESY experiments and methylation data.
(PPT)

Figure S4 **GC-MS analysis of permethylated N-acetylgalactosaminyl-threitol from the fraction II obtained after periodate oxidation of GG.** TIC, total ion chromatogram of permethylated fraction II. CI, chemical ion spectra using NH_4_ as collision gas of the main peak eluted at 21 min. EI, electonic impact spectra of the peak eluted at 21 min. Ion mass m/z were identified according to Fournet et al., [62]. Ion J1 = 207; A1 = 260, A2 = 228, ion [M-NH-MeCOMe] = 350, F1 = 142; H1 = 129; H2 = 87.
(PPT)

Figure S5 **Gel filtration analysis of degraded SGG and PGG fractions of** ***A. fumigatus*** **.** Gel permeation chromatography was performed on a HW40S column eluted with a 0.25% acetic acid solution. A. Analysis of solubilised oligosaccharides obtained after periodate-oxidation of GG. The three carbohydrate containing fractions (I-III) are identified by the refractometry index (RI). B Analysis of solubilised oligosaccharides obtained after nitrous deamination of the GG. Carbohydrates were detected with the phenol-sulfuric method (OD reading at 492 nm). (SGG, urea-soluble GG; PGG, urea-insoluble GG)
(PPT)

Figure S6 **Spearman**'**s representation of the correlation between reactivity of sera from a blood bank against the galactosaminogalactan (GG) of** ***A. fumigatus*** **and a N-glycosylated recombinant protein of** ***Campylobacter jejuni*** **(AcraA).** ELISA ODs obtained with 131 sera against GG (y axis) and AcraA (x axis) showing the fit between these two populations using the JMP software. Bivariate density ellipse with P = 0.95 is shown. Spearman's rho value ρ  =  0.71 (p<0.0001).
(PPT)

Figure S7 **Examples of ELISA inhibition by α1-4GalNAc oligosaccharides of serum reactivity towards PGG of** ***A. fumigatus*** **or AcraA of** ***C. jejuni*** **.** Wells were coated with PGG or AcraA and the serum was incubated with increasing concentration of the GalNAc oligosaccharides obtained by partial HCl hydrolysis of GG. The antibody reactivity to both antigens was inhibited by the linear GalNAc oligosaccharides, indicating that the two antigens share the same epitope in 80% of serum samples. In 20% of sera, the GG recognition was not fully inhibited by the GalNAc oligosaccharides.
(PPT)

Figure S8 **MALDI-TOF mass spectra of the oligosaccharide fraction obtained by partial HCl hydrolysis.** Partial hydrolysis of SGG was performed by 0.1 M HCl at 100°C for 3 h. Solubilised material (G25-I) was purified by gel filtration on a G25 Sephadex column. (mass m/z  =  [M+Na]^+^) GN: N-acetylgalactosamine. G: galactose. The fraction of the hydrolysate excluded from a G25-Sephadex column contained a mixture of oligosaccharides with an average of 7.5 GalNAc per molecule and confirmed the presence of oligoGalNAc in the GG polysaccharide chain. This mild acid hydrolysis was an alternative method to periodate oxidation to prepare quickly and in a single step GalNAc oligosaccharides.
(PPT)

Table S1 **^1^H and ^13^C NMR chemical shifts (ppm) and coupling constants (J_H,H_,J_C,H_ Hz) for the SGG polysaccharidic fraction II obtained after periodate oxidation (** **Fig. 2** **).**
(DOC)

Table S2 **^1^H and ^13^C NMR chemical shifts (ppm) and coupling constants (J_H,H_ ,J_C,H_ Hz) for the SGG polysaccharidic fraction I obtained after nitrous deamination (** **Fig. 3** **).**
(DOC)
