# Supplementary figures and images for: Galactosaminogalactan, a New Immunosuppressive Polysaccharide of Aspergillus fumigatus
Source: PLoS Pathog. 2011 Nov 10;7(11):e1002372. doi: 10.1371/journal.ppat.1002372 (PMC3213105; doi:10.1371/journal.ppat.1002372)

## Slide 1
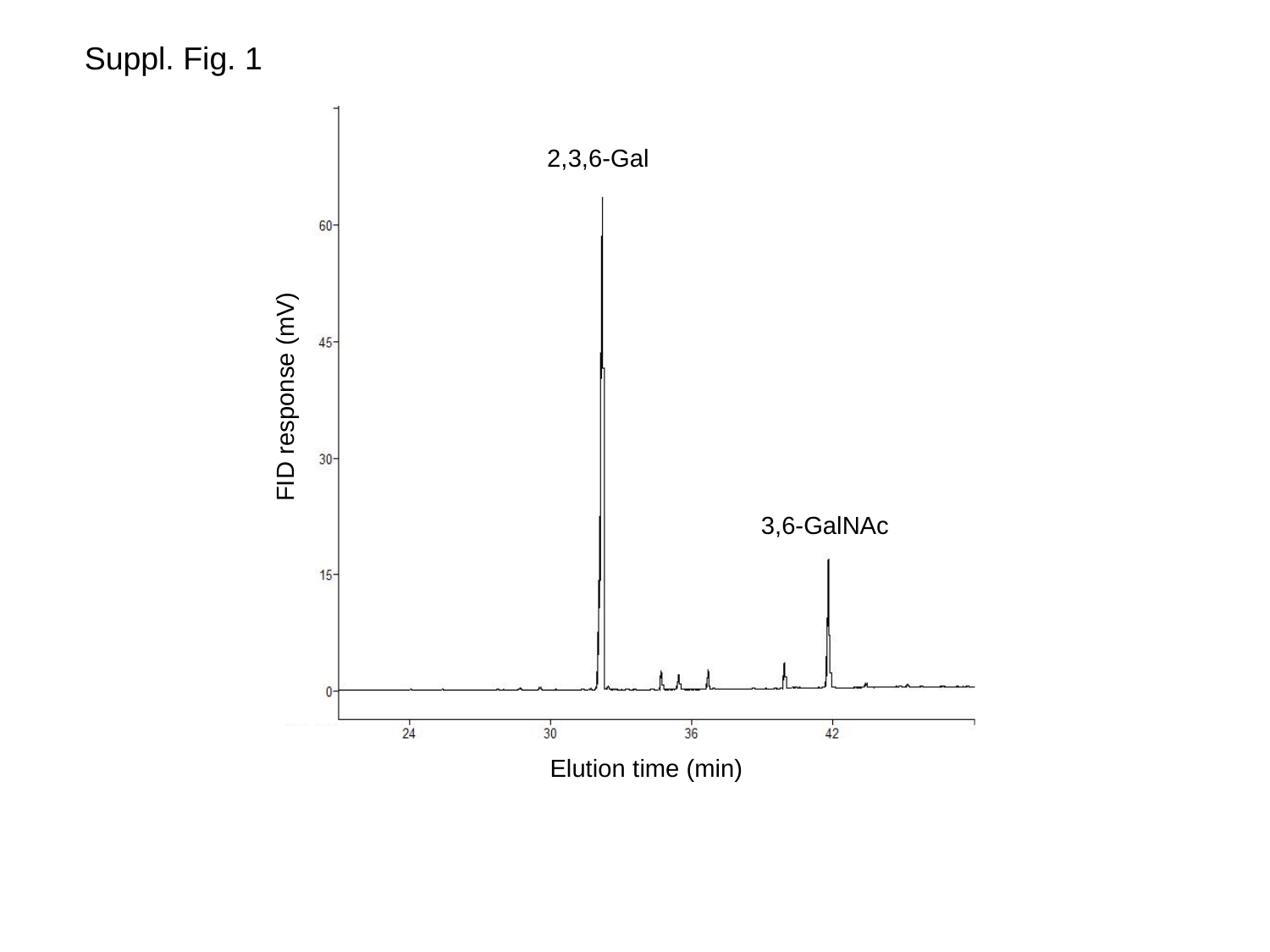

Suppl. Fig. 1
2,3,6-Gal
FID response (mV)
3,6-GalNAc
Elution time (min)

Supplement: Figure S1 — Gas-liquid chromatography of methyl ethers obtained after permethylation of the galactosaminogalactan of A. fumigatus . Methyl ethers (2,3,6-Gal: 2,3,6-tri-O-methyl-1,4,5-tri-O-acetyl-galactitol; 3,6-GalNAc: 3,6-di-O-methyl-1,4,5-tri-O-acetyl-N-methyl-N-acetyl-galactosaminitol) were obtained after hydrolysis, reduction and acetylation of the permethylated GG. (PPT) [file ppat.1002372.s001.ppt]

## Slide 1
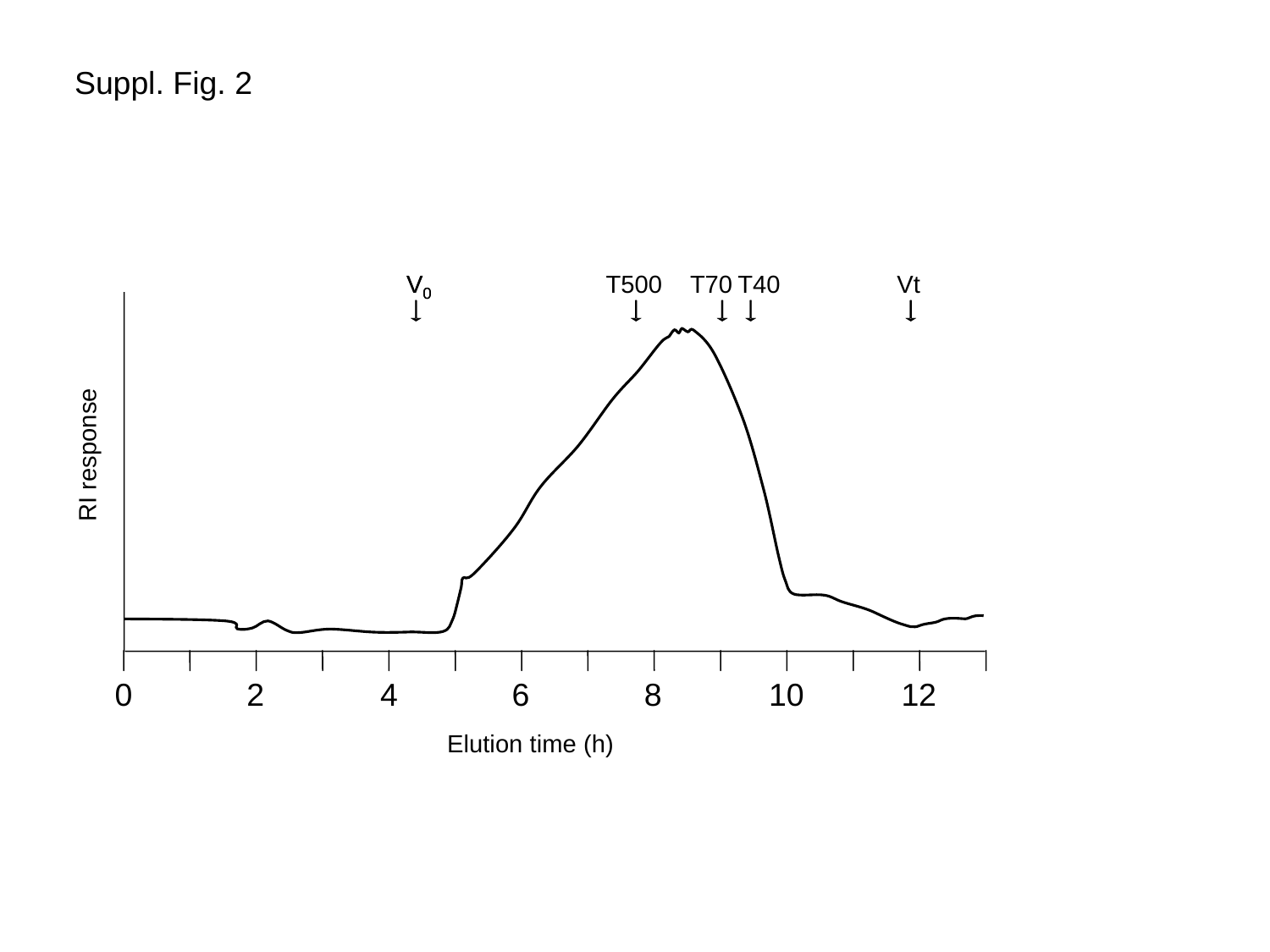

Suppl. Fig. 2
Vt
V
V
T500
T70
T40
0
0
RI response
0
2
4
6
8
10
12
Elution time (h)

Supplement: Figure S2 — Gel permeation analysis of carboxymethylated urea-soluble galactosaminogalactan of A. fumigatus. Gel permeation was performed on a Sephacryl S400 column. Dextrans (Pharmacia, T2000, T500, T70, T40) were used as standards for the column calibration. Fractions were detected by the refractometry index. (PPT) [file ppat.1002372.s002.ppt]

## Slide 1
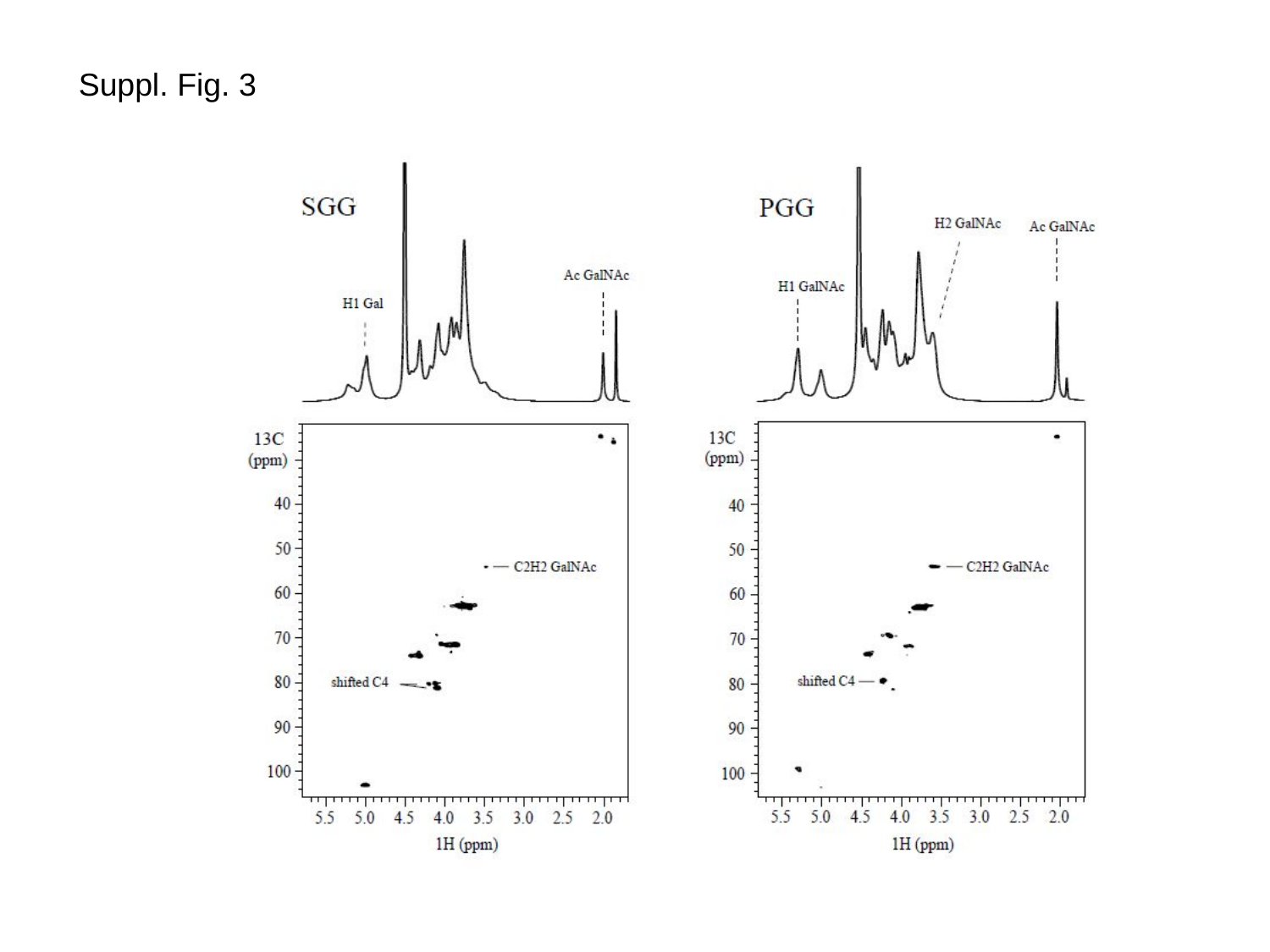

Suppl. Fig. 3

Supplement: Figure S3 — 1D 1H and 2D 1H, 13C HSQC spectrum of carboxymethylated galactosaminogalactan (SGG, urea-soluble GG; PGG, urea-insoluble GG). The 1D 1H and 2D 1H, 13C gHSQC spectra of carboxymethylated GG fractions exhibited two main signals in the sugar anomeric region at 5.003/103.07 and 5.287/99.07 ppm compatible with α-anomers. No β-anomeric configuration was observed. The methyl signal at 2.032/24.61 ppm, characteristic of an acetyl group, and the proton at 5.287 ppm, correlated with the typical upfield shifted H2/C2 (3.587/53.68 ppm) of a 2-N-acetylation, were in agreement with the presence of N-acetylgalactosamine. NMR data showed downfield shifts for the carbone-4 of both sugar residues, indicating their 4-O substitution and their pyranose configuration that was in agreement with the NOESY experiments and methylation data. (PPT) [file ppat.1002372.s003.ppt]

## Slide 1
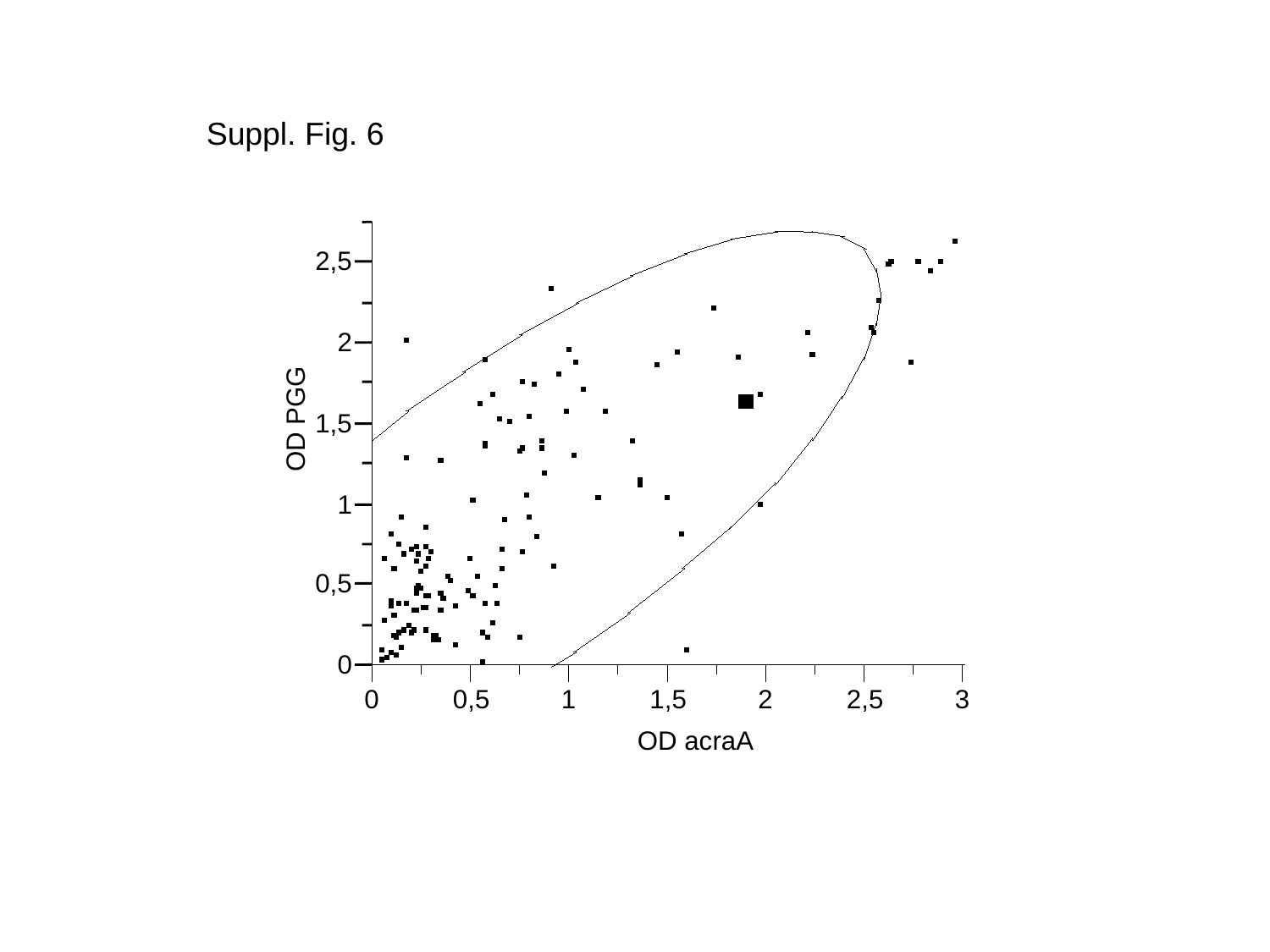

Suppl. Fig. 6
2,5
2
OD PGG
1,5
1
0,5
0
0
0,5
1
1,5
2
2,5
3
OD acraA

Supplement: Figure S6 — Spearman's representation of the correlation between reactivity of sera from a blood bank against the galactosaminogalactan (GG) of A. fumigatus and a N-glycosylated recombinant protein of Campylobacter jejuni (AcraA). ELISA ODs obtained with 131 sera against GG (y axis) and AcraA (x axis) showing the fit between these two populations using the JMP software. Bivariate density ellipse with P = 0.95 is shown. Spearman's rho value ρ = 0.71 (p<0.0001). (PPT) [file ppat.1002372.s006.ppt]

## Slide 1
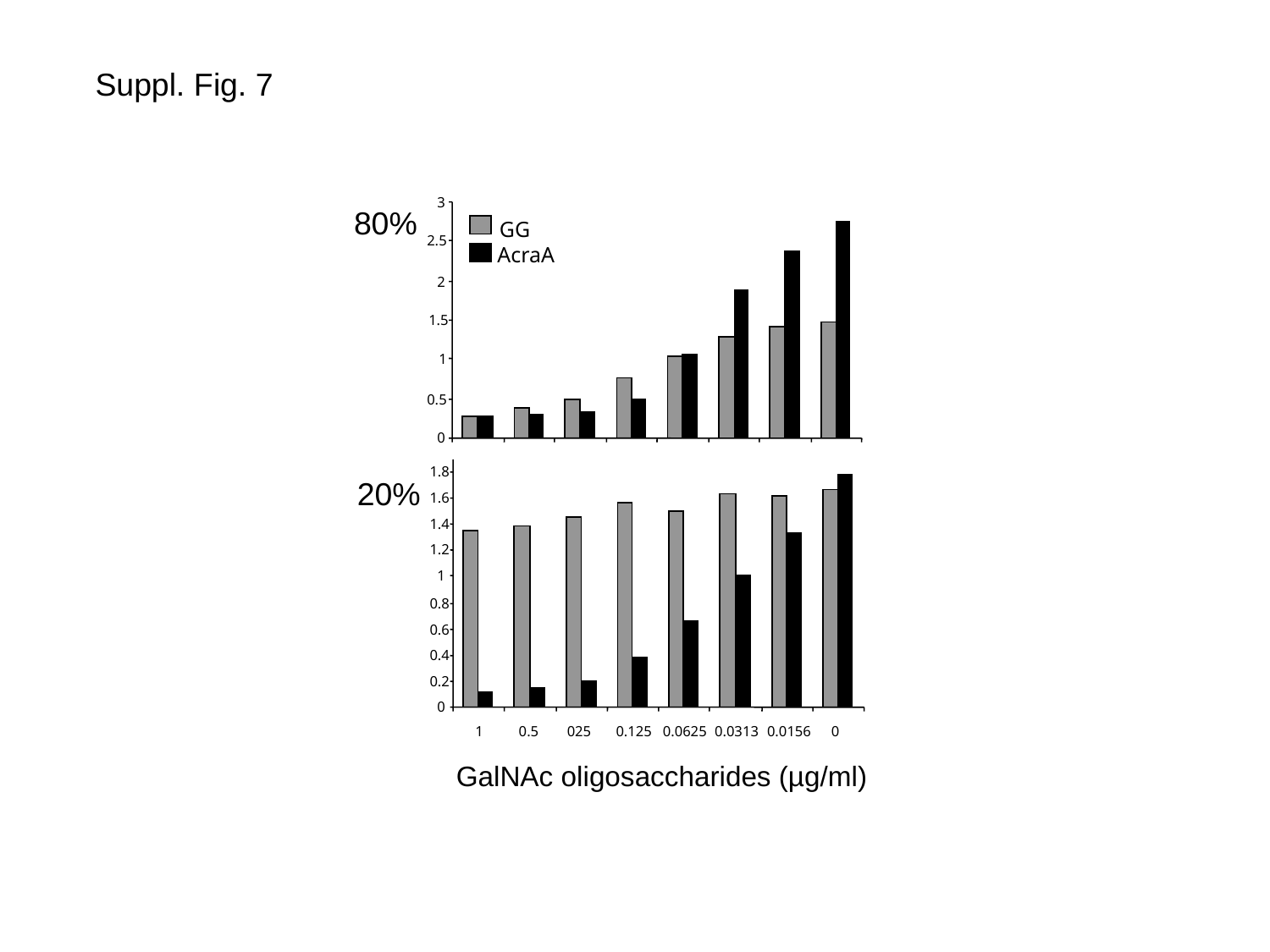

Suppl. Fig. 7
3
GG
AcraA
2.5
2
1.5
1
0.5
0
80%
1.8
1.6
1.4
1.2
1
0.8
0.6
0.4
0.2
0
1
0.5
025
0.125
0.0625
0.0313
0.0156
0
20%
GalNAc oligosaccharides (µg/ml)

Supplement: Figure S7 — Examples of ELISA inhibition by α1-4GalNAc oligosaccharides of serum reactivity towards PGG of A. fumigatus or AcraA of C. jejuni . Wells were coated with PGG or AcraA and the serum was incubated with increasing concentration of the GalNAc oligosaccharides obtained by partial HCl hydrolysis of GG. The antibody reactivity to both antigens was inhibited by the linear GalNAc oligosaccharides, indicating that the two antigens share the same epitope in 80% of serum samples. In 20% of sera, the GG recognition was not fully inhibited by the GalNAc oligosaccharides. (PPT) [file ppat.1002372.s007.ppt]

## Slide 1
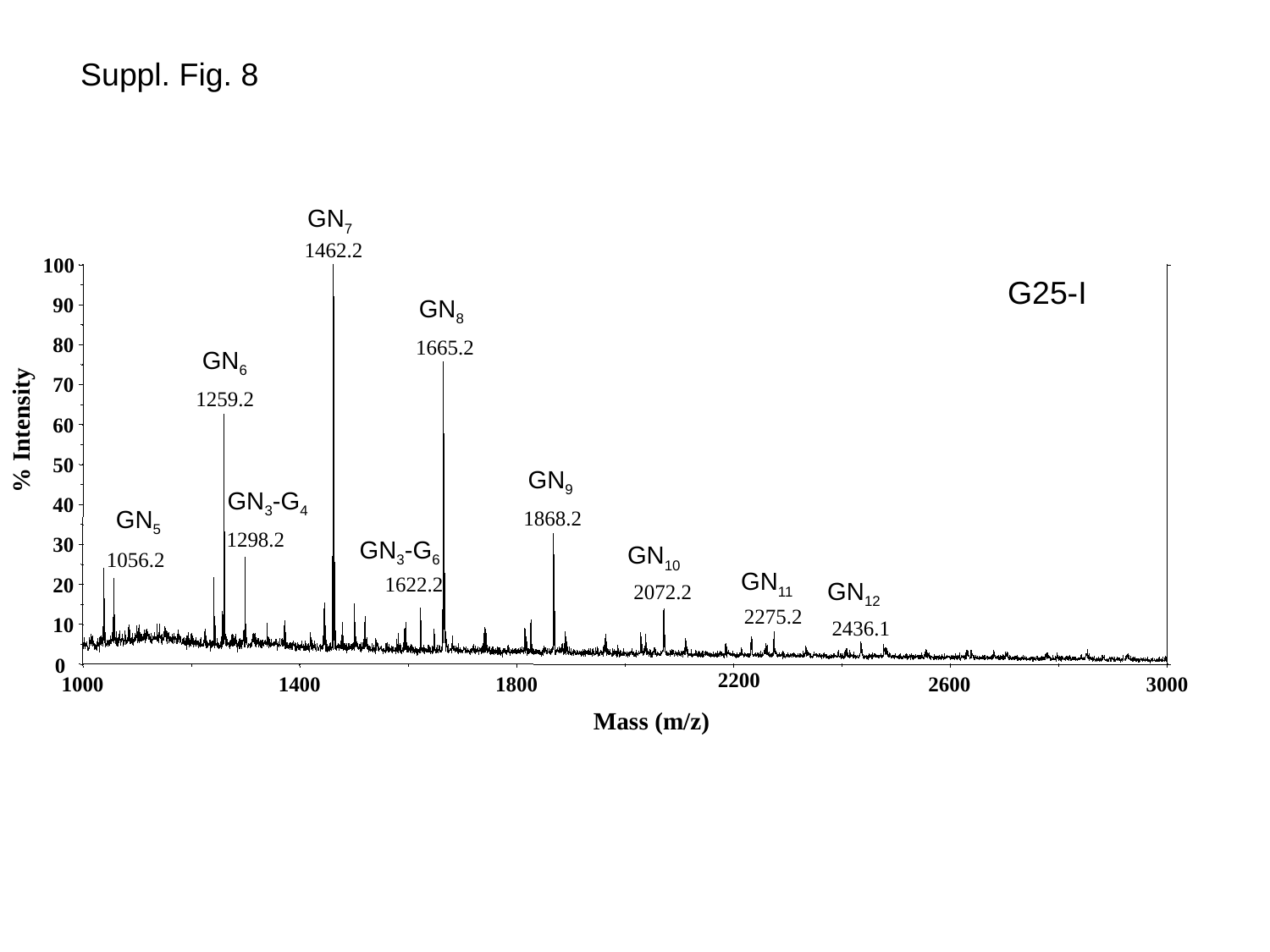

Suppl. Fig. 8
GN7
1462.2
100
GN8
90
80
1665.2
GN6
70
1259.2
60
% Intensity
50
GN9
GN3-G4
40
GN5
1868.2
1298.2
GN3-G6
30
GN10
1056.2
GN11
GN12
1622.2
20
2072.2
2275.2
10
2436.1
0
2200
1000
1400
1800
2600
3000
Mass (m/z)
G25-I

Supplement: Figure S8 — MALDI-TOF mass spectra of the oligosaccharide fraction obtained by partial HCl hydrolysis. Partial hydrolysis of SGG was performed by 0.1 M HCl at 100°C for 3 h. Solubilised material (G25-I) was purified by gel filtration on a G25 Sephadex column. (mass m/z = [M+Na]+) GN: N-acetylgalactosamine. G: galactose. The fraction of the hydrolysate excluded from a G25-Sephadex column contained a mixture of oligosaccharides with an average of 7.5 GalNAc per molecule and confirmed the presence of oligoGalNAc in the GG polysaccharide chain. This mild acid hydrolysis was an alternative method to periodate oxidation to prepare quickly and in a single step GalNAc oligosaccharides. (PPT) [file ppat.1002372.s008.ppt]
