# Supplementary material for: Galactosaminogalactan, a New Immunosuppressive Polysaccharide of Aspergillus fumigatus
Source: PLoS Pathog. 2011 Nov 10;7(11):e1002372. doi: 10.1371/journal.ppat.1002372 (PMC3213105; doi:10.1371/journal.ppat.1002372)
Supplement: Figure S4 — GC-MS analysis of permethylated N-acetylgalactosaminyl-threitol from the fraction II obtained after periodate oxidation of GG. TIC, total ion chromatogram of permethylated fraction II. CI, chemical ion spectra using NH4 as collision gas of the main peak eluted at 21 min. EI, electonic impact spectra of the peak eluted at 21 min. Ion mass m/z were identified according to Fournet et al., [62]. Ion J1 = 207; A1 = 260, A2 = 228, ion [M-NH-MeCOMe] = 350, F1 = 142; H1 = 129; H2 = 87. (PPT) [file ppat.1002372.s004.ppt]

## Slide 1
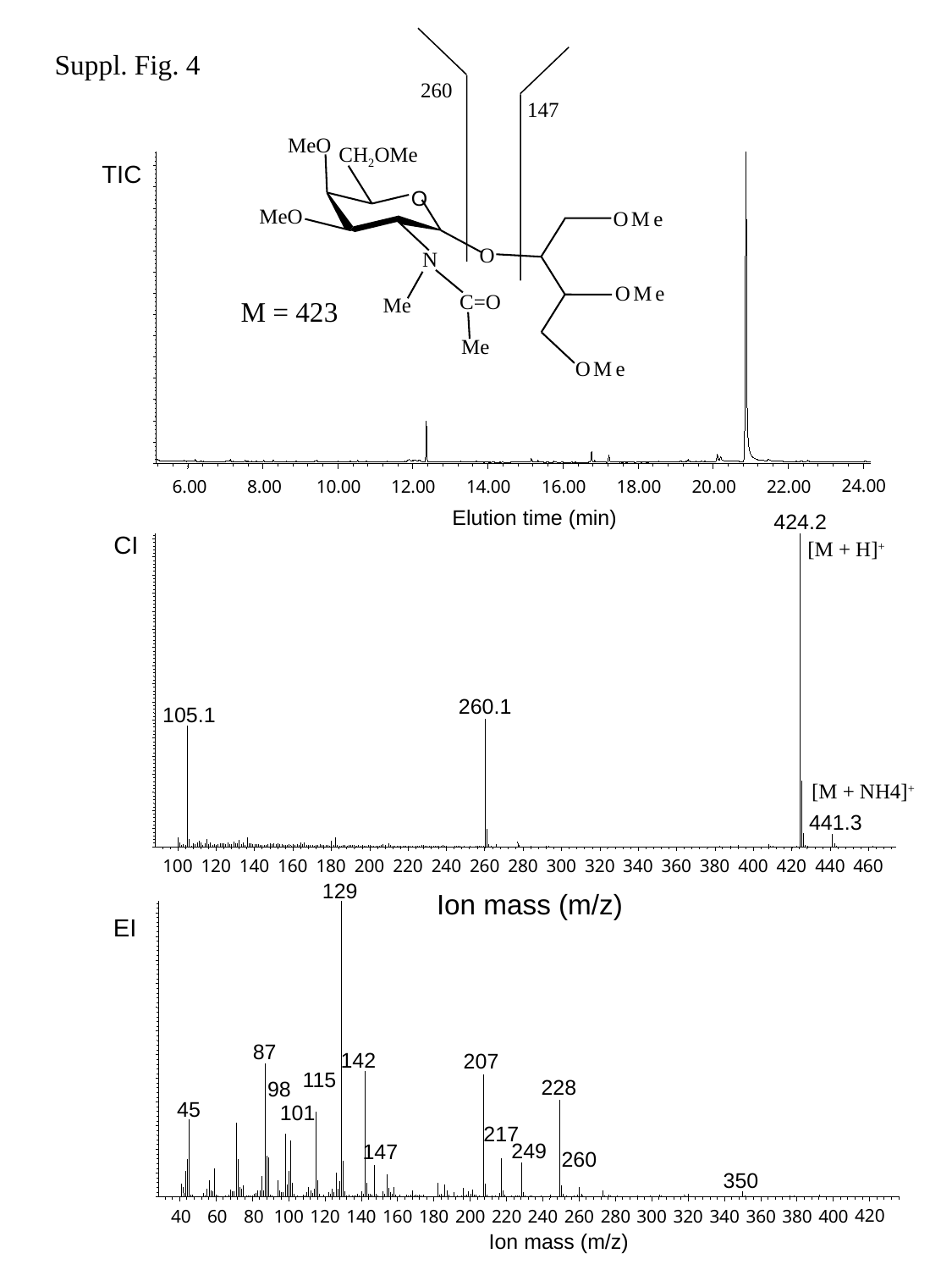

260
147
MeO
CH2OMe
O
MeO
O
M
e
N
O
O
M
e
C=O
Me
Me
O
M
e
Suppl. Fig. 4
24.00
6.00
8.00
10.00
12.00
14.00
16.00
18.00
20.00
22.00
Elution time (min)
TIC
M = 423
424.2
CI
260.1
105.1
441.3
460
100
120
140
160
180
200
220
240
260
280
300
320
340
360
380
400
420
440
[M + H]+
[M + NH4]+
129
EI
87
142
207
115
228
98
45
101
217
249
147
260
350
420
40
60
80
100
120
140
160
180
200
220
240
260
280
300
320
340
360
380
400
Ion mass (m/z)
Ion mass (m/z)
