# Supplementary material for: Galactosaminogalactan, a New Immunosuppressive Polysaccharide of Aspergillus fumigatus
Source: PLoS Pathog. 2011 Nov 10;7(11):e1002372. doi: 10.1371/journal.ppat.1002372 (PMC3213105; doi:10.1371/journal.ppat.1002372)
Supplement: Figure S5 — Gel filtration analysis of degraded SGG and PGG fractions of A. fumigatus . Gel permeation chromatography was performed on a HW40S column eluted with a 0.25% acetic acid solution. A. Analysis of solubilised oligosaccharides obtained after periodate-oxidation of GG. The three carbohydrate containing fractions (I-III) are identified by the refractometry index (RI). B Analysis of solubilised oligosaccharides obtained after nitrous deamination of the GG. Carbohydrates were detected with the phenol-sulfuric method (OD reading at 492 nm). (SGG, urea-soluble GG; PGG, urea-insoluble GG) (PPT) [file ppat.1002372.s005.ppt]

## Slide 1
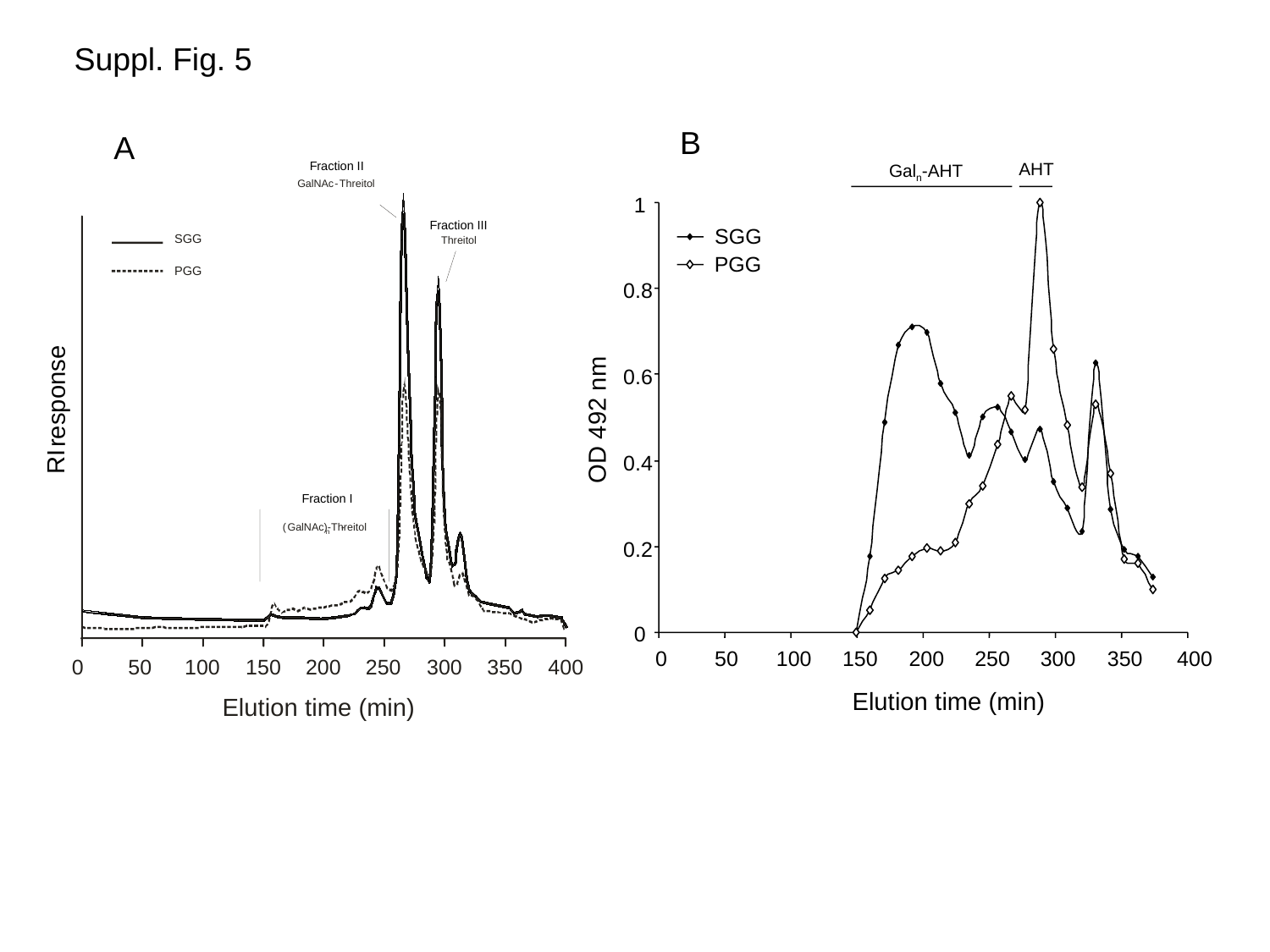

Suppl. Fig. 5
B
A
AHT
Galn-AHT
Fraction II
GalNAc
-
Threitol
1
SGG
Fraction III
SGG
Threitol
PGG
PGG
0.8
0.6
response
OD 492 nm
RI
0.4
Fraction I
(
GalNAc)-Threitol
-
n
0.2
0
0
50
100
150
200
250
300
350
400
0
50
100
150
200
250
300
350
400
Elution time (min)
Elution time (min)
