# Supplementary material for: Galactosaminogalactan, a New Immunosuppressive Polysaccharide of Aspergillus fumigatus
Source: PLoS Pathog. 2011 Nov 10;7(11):e1002372. doi: 10.1371/journal.ppat.1002372 (PMC3213105; doi:10.1371/journal.ppat.1002372)
Supplement: Table S1 — 1H and 13C NMR chemical shifts (ppm) and coupling constants (JH,H,JC,H Hz) for the SGG polysaccharidic fraction II obtained after periodate oxidation ( Fig. 2 ). (DOC) [file ppat.1002372.s009.doc]

**Table S1.** ^1^H and ^13^C NMR chemical shifts (ppm) and coupling constants (J_H,H_ ,J_C,H_ Hz) for the SGG polysaccharidic fraction II obtained after periodate oxidation (Fig. 2).

| α-GalNAc1- | H1 | H2 | H3 | H4 | H5 | H6,6' | CH3 |  |
| --- | --- | --- | --- | --- | --- | --- | --- | --- |
|  | *^3^J_1,2_ (^1^J_C1H1_)* | *^3^J_2,3_* | *^3^J_3,4_* | *^3^J_4,5_* | *^3^J_5,6_ -^3^J_5,6'_* | *^2^J_6,6'_* |  |  |
|  | C1 | C2 | C3 | C4 | C5 | C6 |  | CO |
|  | 5.004 | 4.134 | 3.921 | 3.976 | 4.066 | 3.722 | 2.052 |  |
|  | *4 (171)* | *11* | *5* | *small* |  |  |  |  |
|  | 101.38 | 52.87 | 70.04 | 71.12 | 73.86 | 63.88 | 25.54 | 177.16 |
|  |  |  |  |  |  |  |  |  |
| -2-threitol | H1,1' | H2 | H3 | H4,4' |  |  |  |  |
|  | C1 | C2 | C3 | C4 |  |  |  |  |
|  | 3.752 | 3.682 | 3.876 | 3.642-3.610 |  |  |  |  |
|  | 63.72 | **82.55** | 73.81 | 64.96 |  |  |  |  |

The 2D ^1^H,^13^C gHSQC of the fraction II (Fig. 2) exhibited one main signal in the anomeric region at 5.004/101.38 ppm. The ^1^H and ^13^C chemical shift analysis of the corresponding spin system revealed it was 2-N-acetylated (upfield shifted H2/C2 at 4.124/52.87 ppm and CH_3_ group at 2.052/25.54 ppm reported in the table). Moreover the examination of ^3^J_H,H_ and ^1^J_C1,H1_ values confirmed the presence of a N-acetyl-α-galactosamine residue. Furthermore, in edited gHSQC experiment, four remaining carbon signals of which two correspond to two methylene carbons at 3.752/63.72 and 3.642-3.610/64.96 ppm were assigned to threitol. Dipolar interactions were observed in the ROESY experiment between the anomeric proton of the GalNAc residue and the H2 proton of threitol indicating the following arrangement for the molecule: α-GalNAc1-2-threitol. This was confirmed in the gHMBC experiment by the observation of a H1/C2 correlation between α-GalNAc and threitol and reinforced by the threitol C2 upfield shift at 82.55ppm (in bold in the table).
