# Supplementary material for: Galactosaminogalactan, a New Immunosuppressive Polysaccharide of Aspergillus fumigatus
Source: PLoS Pathog. 2011 Nov 10;7(11):e1002372. doi: 10.1371/journal.ppat.1002372 (PMC3213105; doi:10.1371/journal.ppat.1002372)
Supplement: Table S2 — 1H and 13C NMR chemical shifts (ppm) and coupling constants (JH,H ,JC,H Hz) for the SGG polysaccharidic fraction I obtained after nitrous deamination ( Fig. 3 ). (DOC) [file ppat.1002372.s010.doc]

**Table S2.** ^1^H and ^13^C NMR chemical shifts (ppm) and coupling constants (J_H,H_ ,J_C,H_ Hz) for the SGG polysaccharidic fraction I obtained after nitrous deamination (Fig. 3).

|  | H1 | H2 | H3 | H4 | H5 | H6,6' |
| --- | --- | --- | --- | --- | --- | --- |
|  | *^3^J_1,2_ (^1^J_C1H1_)* | *^3^J_2,3_* | *^3^J_3,4_* | *^3^J_4,5_* |  |  |
|  | C1 | C2 | C3 | C4 | C5 | C6 |
| α-Galp1-4-AHT | |  |  |  |  |  |
|  |  |  |  |  |  |  |
| α-Gal1- | 5.065 | 3.84 | 3.95 | 3.993 | 4.219 | 3.73 |
|  | *3.5 (170,3)* | *8.2* | *small* | *small* |  |  |
|  | 101.82 | 71.3 | 71.54 | 71.54 | 73.88 | 63.76 |
|  |  |  |  |  |  |  |
| -4-AHT | 5.017 | 3.852 | 4.342 | 4.441 | 4.255 | 3.806 |
|  | 4.7 |  |  |  |  |  |
|  | 92.21 | 86.76 | 74.45 | **80.19** | 82.59 | 62.54 |
|  |  |  |  |  |  |  |
| α-Gal1[-4-α-Gal1]_n_-4-α-Gal1-4-AHT | | | | |  |  |
|  |  |  |  |  |  |  |
| α-Gal1- | 4.997 | 3.837 | 3.998 | 4.016 | 4.308 | 3.704 |
|  | 3.8 | 10.5 | small | small |  |  |
|  | 103.17 | 71.3 | 71.54 | 71.66 | 73.74 | 63.29 |
|  |  |  |  |  |  |  |
| [−4-α-Gal1-]_n_ | 5.028 | 3.889 | 3.976 | 4.129 | 4.386 | 3.74 |
| n ≥ 0 | *3.5 (170,2)* | *10.6* | *small* | *small* |  |  |
|  | 103.14 | 71.54 | 71.54 | **81.27** | 73.82 | 62.65 |
|  |  |  |  |  |  |  |
| -4-α-Gal1- | 5.108 | 3.903 | 4.039 | 4.105 | 4.279 | 3.83 |
|  | *3.9 (172,0)* | *9.2* | *small* | *small* |  |  |
|  | 101.92 | 71.54 | 71.51 | **81.38** | 74.21 | 62.93 |
|  |  |  |  |  |  |  |
| -4-AHT | 5.017 | 3.852 | 4.342 | 4.441 | 4.255 | 3.806 |
|  | 4.7 |  |  |  |  |  |
|  | 92.21 | 86.76 | 74.45 | **80.19** | 82.59 | 62.54 |

The 2D ^1^H,^13^C gHSQC anomeric region of this fraction showed 3 main signals at 4.997/103.17, 5.028/103.14 and 5.108/101.92 ppm and a minor signal at 5.065/101.82 ppm that all were assigned to α-galactose residues. Galactose residues with H1/C1 at 4.997/103.17 and 5.065/101.82 ppm were identified at non-reducing end from the absence of shifted resonances for other nuclei of these moieties. The 5.017/92.21 ppm signals were identified as H1/C1 of reducing end 2,5-anhydrotalose (AHT) residue present in its hydrated form. A ROESY experiment showed strong correlations between H1 of the non reducing end galactose at 4.997 ppm and H4 of galactose residues at 4.105 and 4.129 ppm. Moreover, H1/C4 correlations between these residues were observed in the gHMBC experiment, indicating the following branched sequence: α-Gal-(1→4)-α-Gal. This is in agreement with C4 downfield chemical shifts at 81.27 and 81.38 ppm observed for the branched galactose residues (in bold in the table). Similarly strong interactions were observed in the ROESY experiment between the branched residue H1 at 5.028 ppm and H4 of galactose residues at 4.105 and 4.129 ppm suggesting the presence of α-Gal1-4-α-Gal1-4-α-Gal sequences that was confirmed by the correlations H1/C4 (5,028/81.27 and 81.38 ppm) in gHMBC experiment. A strong interaction was also observed in the ROESY experiment between the branched residue H1 at 5.108 ppm and H4 of AHT residue at 4.441 ppm. Moreover, C1/H4 and H1/C4 correlations between these residues were observed in the gHMBC experiment, indicating the following linkage: -4-α-Gal1-4-AHT. This sequence accounts for the C4 downfield chemical shift at 80.19 ppm of AHT (in bold in the table).
